# SGIP1 binding to the α-helical H9 domain of cannabinoid receptor 1 promotes axonal surface expression

**DOI:** 10.1242/jcs.261551

**Published:** 2024-06-12

**Authors:** Alexandra Fletcher-Jones, Ellen Spackman, Tim J. Craig, Yasuko Nakamura, Kevin A. Wilkinson, Jeremy M. Henley

**Affiliations:** ^1^ School of Biochemistry, Centre for Synaptic Plasticity, University of Bristol, Biomedical Sciences Building, Bristol, BS8 1TD, UK; ^2^ School of Applied Sciences, University of the West of England, Coldharbour Lane, Bristol, BS16 1QY, UK; ^3^ School of Physiology, Pharmacology and Neuroscience, Centre for Synaptic Plasticity, University of Bristol, Biomedical Sciences Building, Bristol, BS8 1TD, UK

**Keywords:** SH3GL interacting endocytic adaptor 1, SGIP1, Cannabinoid receptor 1, Endocannabinoid system, Endocytosis, Presynapse, Synaptic transmission

## Abstract

Endocannabinoid signalling mediated by cannabinoid receptor 1 (CB1R, also known as CNR1) is critical for homeostatic neuromodulation of both excitatory and inhibitory synapses. This requires highly polarised axonal surface expression of CB1R, but how this is achieved remains unclear. We previously reported that the α-helical H9 domain in the intracellular C terminus of CB1R contributes to axonal surface expression by an unknown mechanism. Here, we show in rat primary neuronal cultures that the H9 domain binds to the endocytic adaptor protein SGIP1 to promote CB1R expression in the axonal membrane. Overexpression of SGIP1 increases CB1R axonal surface localisation but has no effect on CB1R lacking the H9 domain (CB1R^ΔH9^). Conversely, SGIP1 knockdown reduces axonal surface expression of CB1R but does not affect CB1R^ΔH9^. Furthermore, SGIP1 knockdown diminishes CB1R-mediated inhibition of presynaptic Ca^2+^ influx in response to neuronal activity. Taken together, these data advance mechanistic understanding of endocannabinoid signalling by demonstrating that SGIP1 interaction with the H9 domain underpins axonal CB1R surface expression to regulate presynaptic responsiveness.

## Teaser

**Summary:** The cannabinoid type 1 receptor is stabilised at the axonal surface through interaction of its intracellular helix 9 domain with the endocytic adaptor protein SGIP1.

## INTRODUCTION

The endocannabinoid system (ECS) is a negative feedback system that homeostatically controls neurotransmission in the brain. By mediating activity-dependent suppression of presynaptic release, the ECS modulates synaptic strength and plasticity, which are fundamental for many brain processes including cognition, appetite, energy expenditure, and learning and memory (Castillo et al., 2012). Moreover, the ECS plays key roles in attenuating stress-induced glutamate release and is implicated in a wide range of neurological and neurodegenerative diseases (Katona and Freund, 2008; Russo, 2018).

Because the pharmacology of the ECS is complex and pleiotropic, drugs that act directly on the system often result in unwanted neurological and psychoactive side effects (Busquets-Garcia et al., 2018). Given these limitations, increased understanding of the biochemistry and cell biology of the ECS could provide new avenues for therapeutic intervention.

In neurons, the main ECS receptor, cannabinoid receptor 1 (CB1R, also known as CNR1), is located predominantly at the axonal membrane (Coutts et al., 2001; Fletcher-Jones et al., 2019; Irving et al., 2000; Leterrier et al., 2006; McDonald et al., 2007a; Rozenfeld and Devi, 2008; Saez et al., 2020; Simon et al., 2013; Thibault et al., 2013; Wickert et al., 2018), particularly at the presynaptic terminal (Dudok et al., 2015; Katona et al., 1999; Nyiri et al., 2005). CB1R activation by endocannabinoids released from the postsynaptic membrane suppresses presynaptic neurotransmitter release via G protein-mediated inhibition of presynaptic voltage-gated Ca^2+^ channels (Mackie and Hille, 1992) and/or adenylyl cyclase activity (Chevaleyre et al., 2007). Thus, the selective targeting of CB1R to the axonal membrane is crucial to its role in regulating activity at the presynapse, yet how this is orchestrated at a molecular level is poorly defined (Fletcher-Jones et al., 2020).

We have reported previously that CB1R is preferentially and directly targeted to axons through the secretory pathway and that polarity is maintained, at least in part, by CB1R being more rapidly endocytosed from the somatodendritic membrane than from the axonal membrane (Fletcher-Jones et al., 2019). Furthermore, we have shown that the 21-residue putative α-helical H9 domain in the intracellular C terminal domain of CB1R (ctCB1R) contributes to the delivery and stabilisation of axonal CB1R (Fletcher-Jones et al., 2019). However, despite this progress, exactly how the H9 domain promotes the axonal surface distribution of CB1R remains to be determined.

SH3-containing GRB2-like protein 3-interacting protein 1 (SGIP1) is abundantly expressed in brain (Trevaskis et al., 2005) and preferentially localises to axons and presynaptic terminals (Hajkova et al., 2016; Lee et al., 2019b; Wilhelm et al., 2014). SGIP1 is an endocytic adaptor protein that has been linked to clathrin-mediated endocytosis (Dergai et al., 2010; Li et al., 2011; Mishra et al., 2021; Stimpson et al., 2009; Uezu et al., 2007; Zhang et al., 2018); however, its precise roles remain elusive and might be isoform-dependent since the longer, less abundant isoform SGIP1α is capable of membrane tubulation, whereas SGIP1 itself is not (Lee et al., 2021).

SGIP1 has been reported to bind ctCB1R in a yeast two-hybrid study, but the site of interaction on CB1R was not determined (Hajkova et al., 2016). Expression studies in HEK293 cells have suggested that SGIP1 interferes with agonist-induced internalisation of CB1R and modulates the recruitment of β-arrestin2 and GRK3, as well as downstream signalling via ERK1 and ERK2 (MAPK3 and MAPK1, respectively; collectively referred to as ERK1/2) (Durydivka et al., 2024, 2023; Gazdarica et al., 2022; Hajkova et al., 2016). Moreover, SGIP1-knockout mice display disrupted ECS-dependent behaviours and altered responses to Δ^9^-tetrahydrocannabinol (THC), including reduced anxiety, reduced acute nociception and increased sensitivity to cannabinoid-induced analgesia, while working memory and exploration remain unaltered (Durydivka et al., 2023; Dvorakova et al., 2021).

Here, we report that SGIP1 binds to the CB1R α-helical H9 domain and acts to stabilise CB1R at the presynaptic membrane. We show that overexpression of SGIP1 increases levels of CB1R at the axonal plasma membrane, whereas SGIP1 knockdown phenocopies the decreased surface expression observed upon deletion of the H9 domain (CB1R^ΔH9^) and impairs CB1R-mediated modulation of synaptic transmission. These data advance mechanistic understanding of how CB1R polarity is established and maintained by identifying SGIP1 as an important mediator of CB1R axonal surface expression. Moreover, these findings open the possibility that manipulating this interaction could be used to regulate the availability of presynaptic CB1R for potential therapeutic benefits.

## RESULTS

### Cloning of SGIP1β from rat cortical neuronal cultures

Deletion of the H9 domain reduces CB1R surface expression and increases CB1R endocytosis in primary neurons (Fletcher-Jones et al., 2019), whereas co-expression of SGIP1 enhances CB1R surface expression in HEK293 cells (Hajkova et al., 2016). Based on these observations we wondered whether SGIP1 interacts with the H9 domain to regulate CB1R surface expression. To investigate this possibility, we amplified rat SGIP1 from cDNA derived from mRNA extracted from primary cortical neurons at 21 days *in vitro* (DIV) and subcloned it into a modified pcDNA3.1 vector to incorporate an N-terminal FLAG tag.

**Fig. 1.**
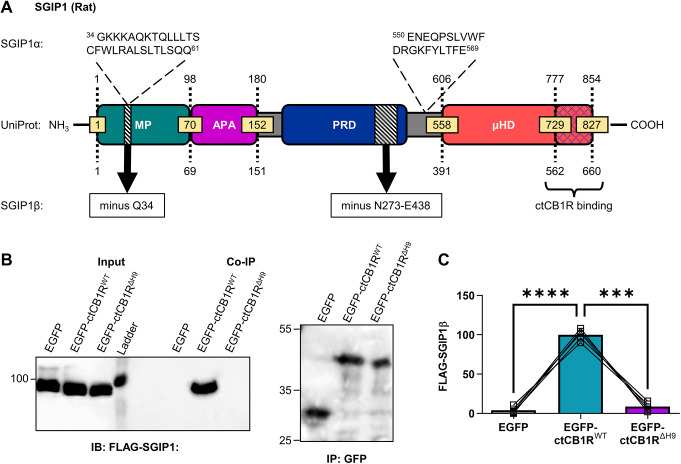
The H9 domain **interacts with SGIP1.** (A) Schematic comparing the three known SGIP1 isoforms. The SGIP1 isoform we cloned from a cDNA library extracted from rat primary cortical cultures, designated here as SGIP1β, conforms to predicted SGIP1 transcript variant X19 (NCBI reference sequence XM_017593774.2). This variant comprises 660 amino acids and differs from the standard SGIP1 variant found on UniProt (which corresponds to predicted transcript variant X9, NCBI reference sequence XM_017593764.2) by two deletions – a single residue deletion (Q34) in the MP domain and a 165-residue deletion (N273–E438) in the PRD (black hatched regions). Importantly, SGIP1β does not contain the two additional regions found in SGIP1α (shown above the UniProt variant diagram), the first of which has been found to be necessary for membrane tubulation (Lee et al., 2021). The blue cross-hatched region in the µ homology domain (µHD) indicates the region that binds ctCB1R (Hajkova et al., 2016). APA, AP-2 activator motif. (B) Representative immunoblots showing co-immunoprecipitation (co-IP) of FLAG–SGIP1β with EGFP–ctCB1R^WT^, but not with EGFP–ctCB1R^ΔH9^ or the EGFP control, in lysates from HEK293T cells. Left: anti-FLAG immunoblot (IB) showing FLAG–SGIP1β in input (5%) and GFP immunoprecipitation (IP) samples. Right: anti-GFP immunoblot of GFP IP samples. Positions of molecular mass markers are indicated in kDa. (C) Quantification of data represented in B. Significantly more FLAG–SGIP1β co-immunoprecipitates with EGFP–ctCB1R^WT^ than with an EGFP control (EGFP versus EGFP–ctCB1R^WT^: mean±s.e.m., 4.04±1.95 versus 100±3.23; ****P<0.0001) or with EGFP–ctCB1R^ΔH9^ (EGFP–ctCB1R^WT^ versus EGFP–ctCB1R^ΔH9^: mean±s.e.m., 100±3.23 versus 8.70±2.38; ***P=0.0002), suggesting that FLAG–SGIP1β specifically interacts with the H9 domain. Level of FLAG–SGIP1β co-immunoprecipitation with EGFP–ctCB1R^ΔH9^ was comparable to that with the EGFP control, suggesting that the H9 domain is the only interaction site of FLAG–SGIP1β in ctCB1R (EGFP versus EGFP–ctCB1R^ΔH9^: mean±s.e.m., 4.04±1.95 versus 8.70±2.38; *P*=0.3340). FLAG–SGIP1β signal was normalised to the GFP IP signal and expressed as a percentage of EGFP–ctCB1R^WT^. Matched one-way ANOVA with Tukey's post hoc test. *n*=5 independent experiments per condition.

The isolated sequence corresponded to predicted SGIP1 transcript variant X19 (NCBI reference sequence XM_017593774.2; Fig. 1A). This 660-amino-acid variant differs from the full-length canonical UniProt entry (transcript variant X9, NCBI reference sequence XM_017593764.2) by two deletions: a single residue deletion in the membrane phospholipid-binding domain (MP domain; Q34) and a 165-residue deletion in the proline-rich domain (PRD). Importantly, this variant, which we refer to as SGIP1β, does not contain the additional sequence found in the longer isoform SGIP1α (NCBI reference sequence NM_001376936.1; transcript variant X1, NCBI reference sequence XM_039109919.1; transcript variant X2, NCBI reference sequence XM_039109920.1) that is necessary for membrane tubulation (Lee et al., 2021). Furthermore, the 99 C-terminal residues D708–N806 in both mouse SGIP1 and rat SGIP1β, which have 100% sequence identity and contain the CB1R-binding domain (Hajkova et al., 2016), are unchanged.

### SGIP1β interacts with the H9 domain of CB1R

To determine whether SGIP1 binds CB1R via the H9 domain*,* we co-transfected HEK293T cells with FLAG-tagged SGIP1β (FLAG–SGIP1β) and either EGFP, EGFP-tagged wild-type ctCB1R (EGFP–ctCB1R^WT^) or EGFP-tagged ctCB1R lacking the H9 domain (EGFP–ctCB1R^ΔH9^). Using GFP-Trap, FLAG–SGIP1β co-immunoprecipitated with EGFP–ctCB1R^WT^ but not with EGFP–ctCB1R^ΔH9^ or the EGFP control, indicating that the H9 domain is required for CB1R binding to SGIP1 (Fig. 1B,C).

### Expression of SGIP1 increases surface expression of wild-type CB1R but not CB1R^ΔH9^

**Fig. 2.**
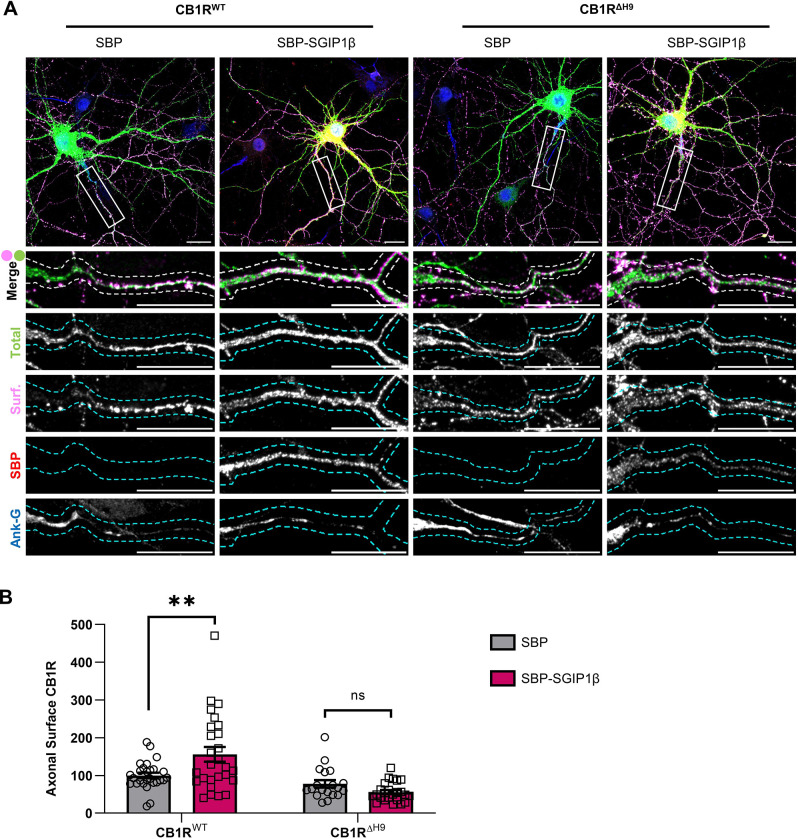
**Overexpression of SGIP1β increases surface expression of CB1R^WT^ but not CB1R^ΔH9^.** (A) Representative confocal images of DIV14 hippocampal neurons expressing EGFP–CB1R^WT^ or EGFP–CB1R^ΔH9^ and either SBP or SBP–SGIP1β. Cells were transfected at DIV12, incubated for a further 2 days, and then surface stained with anti-GFP antibody. Upper panels for each condition show a whole-cell field of view, and lower panels are enlargements of the axonal ROIs indicated by boxes. Green, total GFP staining; magenta, surface GFP staining (Surf); red, SBP–SGIP1β (SBP); blue, axon marker (ankyrin-G, Ank-G). Merge panels show total and surface GFP staining. Dashed lines indicate example processes that were analysed. Scale bars: 20 μm. See Fig. S2 for enlargements and quantification of dendritic ROIs. (B) Quantification of data represented in A. SGIP1β overexpression causes a significant increase in surface expression of EGFP–CB1R^WT^ in axons (CB1R^WT^ with SBP versus CB1R^WT^ with SBP–SGIP1β: mean±s.e.m., 100.00±7.09 versus 156.06±19.43; *n*=27 versus *n*=27; **P=0.0022). SGIP1β overexpression did not alter surface expression of EGFP–CB1R^ΔH9^ (CB1R^ΔH9^ with SBP versus CB1R^ΔH9^ with SBP–SGIP1β: mean±s.e.m., 77.92±9.65 versus 55.90±5.20; *n*=19 versus *n*=24; *P*=0.4281). Surface fluorescence was normalised to total fluorescence and is shown as a percentage of the CB1R^WT^ with SBP control. Two-way ANOVA with Sidak's post hoc test; *n*=19–27 neurons from four independent neuronal cultures per condition. ns, not significant.

To determine the role of SGIP1 on CB1R axonal surface localisation, we co-transfected DIV12 neurons with EGFP-tagged wild-type full-length CB1R (EGFP–CB1R^WT^) or EGFP-tagged CB1R^ΔH9^ (EGFP–CB1R^ΔH9^), and with either a streptavidin-binding peptide tag (SBP) control or SBP-tagged SGIP1β (SBP–SGIP1β). Following transfection, neurons were incubated for a further 2 days and then stained for surface CB1R using anti-GFP antibody (Fig. 2A). Co-expression of SBP–SGIP1β, but not of the SBP control, significantly increased axonal surface levels of EGFP–CB1R^WT^ (Fig. 2B), comparable to what occurs in HEK293 cells (Hajkova et al., 2016). Importantly, no such increase was observed for EGFP–CB1R^ΔH9^, suggesting that the H9 domain is necessary for this effect to occur (Fig. 2B).

### SGIP1 knockdown reduces surface expression of CB1R^WT^ but not CB1R^ΔH9^

**Fig. 3.**
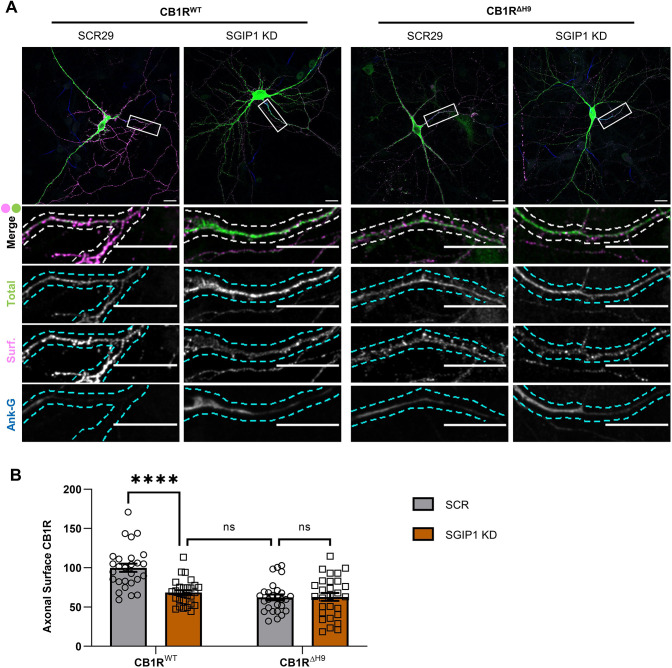
**SGIP1 knockdown reduces surface expression of CB1R^WT^ but not CB1R^ΔH9^.** (A) Representative confocal images of DIV14 hippocampal neurons expressing EGFP–CB1R^WT^ or EGFP–CB1R^ΔH9^ and either a 25-mer shRNA targeting SGIP1 (SGIP1 KD) or a non-targeting scrambled 29-mer shRNA control (SCR29). Cells were transfected at DIV9 and left for 5 days to ensure knockdown then surface stained with anti-GFP antibody. Upper panels for each condition show a whole-cell field of view, and lower panels are enlargements of the axonal ROIs indicated by boxes. Green, total GFP staining; magenta, surface GFP staining (Surf); blue, axon marker (ankyrin-G, Ank-G). Merge panels show total and surface GFP staining. Dashed lines indicate example processes that were analysed. Scale bars: 20 μm. See Fig. S3 for enlargements and quantification of dendritic ROIs. (B) Quantification of data represented in A. SGIP1 knockdown causes a significant reduction in axonal surface expression of EGFP–CB1R^WT^ (CB1R^WT^ with SCR29 versus CB1R^WT^ with SGIP1 KD: mean±s.e.m., 100±5.15 versus 68.46±3.04; *n*=27 versus *n*=28; ****P<0.0001), which phenocopies the reduced surface expression phenotype of EGFP–CB1R^ΔH9^ (CB1R^WT^ with SGIP1 KD versus CB1R^ΔH9^ with SCR29: mean±s.e.m., 68.46±3.04 versus 62.31±3.73; *n*=28 versus *n*=27; *P*=0.896). The effect of SGIP1 KD is occluded for EGFP–CB1R^ΔH9^ (CB1R^ΔH9^ with SCR29 versus CB1R^ΔH9^ with SGIP1 KD: mean±s.e.m., 62.31±3.73 versus 62.88±4.93; *n*=27 versus *n*=28; *P*> 0.999). Surface fluorescence was normalised to total fluorescence and is shown as a percentage of CB1R^WT^ with SCR29. Two-way ANOVA with Sidak's post hoc test. *n*=27–28 neurons from five independent neuronal cultures per condition. ns, not significant.

Next, we transfected DIV9 hippocampal neurons with a scrambled shRNA (SCR29) or an shRNA knockdown construct that targets all known isoforms of SGIP1 (Uezu et al., 2007), and with either EGFP–CB1R^WT^ or EGFP–CB1R^ΔH9^ (Fig. S1). Following transfection, neurons were incubated for a further 5 days to ensure complete knockdown and were then live stained for surface CB1R using an anti-GFP antibody (Fig. 3A). Consistent with a role for SGIP1 in promoting CB1R axonal surface expression, SGIP1 knockdown reduced surface EGFP–CB1R^WT^ in axons to levels equivalent to those of EGFP–CB1R^ΔH9^ (Fig. 3B). Importantly, SGIP1 knockdown did not further reduce surface expression of EGFP–CB1R^ΔH9^ (Fig. 3B). These data demonstrate that CB1R^ΔH9^ is insensitive to regulation by SGIP1 and strongly suggest that the reduced surface expression phenotype of EGFP–CB1R^ΔH9^ is due to an inability to bind SGIP1.

We, and others, have shown that although CB1R is delivered to the dendritic plasma membrane, it is rapidly internalised (Coutts et al., 2001; Fletcher-Jones et al., 2019; Leterrier et al., 2006; McDonald et al., 2007b; Simon et al., 2013). Although SGIP1 has been reported to preferentially localise to axons and presynaptic terminals (Hajkova et al., 2016; Lee et al., 2019a; Wilhelm et al., 2014), SBP–SGIP1β appeared to be present throughout the neuron. However, whereas no effect of SBP–SGIP1β expression on dendritic CB1R surface localisation was detected (Fig. S2A,B), pan-SGIP1 knockdown reduced dendritic surface levels of CB1R^WT^ but not CB1R^ΔH9^ (Fig. S3A,B). These results raise the possibility that an isoform other than SGIP1β might affect dendritic CB1R surface localisation. Interestingly, however, neither SBP–SGIP1β overexpression nor pan-SGIP1 knockdown affected surface polarity (A/D ratio; Figs S2C, S3C), suggesting that SGIP1 acts to stabilise CB1R surface expression in both axons and dendrites.

### SGIP1 knockdown increases CB1R accumulation in somatic endolysosomes

**Fig. 4.**
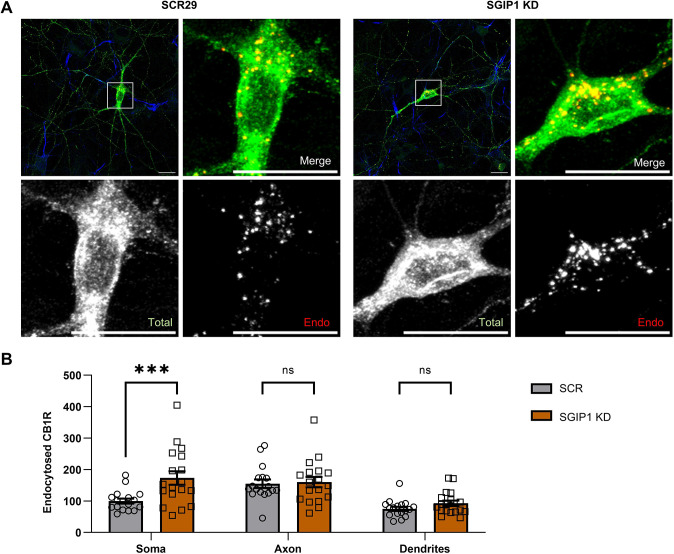
**SGIP1 knockdown increases CB1R accumulation at somatic endolysosomes after agonist stimulation.** (A) Representative confocal images of DIV14 hippocampal neurons expressing EGFP–CB1R^WT^ and either an shRNA targeting SGIP1 (SGIP1 KD) or a non-targeting scrambled control (SCR29). Cells were transfected at DIV9 and left for 5 days to ensure knockdown. Cells were pre-incubated in leupeptin for 3 h to block degradation. Surface EGFP–CB1R was then labelled with anti-GFP antibody, and endocytosis was induced by incubation in 5 µM 2-AG for 1 h. Residual surface anti-GFP was stripped off prior to fixation and staining to reveal the endocytosed pool of receptors. Upper left panels for each condition show a whole-cell field of view, and additional panels are enlargements of soma (indicated by boxes). Green, total GFP staining; red, endocytosed GFP staining (Endo); blue, axon marker (ankyrin-G). Scale bars: 20 μm. See Fig. S4 for enlargements and quantification of axonal and dendritic ROIs. (B) Quantification of data represented in A. Knockdown of SGIP1 leads to accumulation of CB1R in the somatic endolysosomal system (SCR29 versus SGIP1 KD: mean±s.e.m., 100.00±8.00 versus 173.21±21.31; *n*=17 versus *n*=18; ***P=0.0008). There was no significant difference in endocytosed CB1R in axons (SCR29 versus SGIP1 KD: mean±s.e.m., 155.19±13.33 versus 160.19±16.52; *n*=17 versus *n*=18; *P*=0.9915) or dendrites (SCR29 versus SGIP1 KD: mean±s.e.m., 75.00±6.65 versus 92.80±8.75; *n*=17 versus *n*=18; *P*=0.7372). Endocytosed fluorescence was normalised to total fluorescence and is shown as a percentage of soma SCR29. Two-way ANOVA with Sidak's post hoc test. *n*=17–18 neurons from five independent neuronal cultures per condition. ns, not significant.

To test whether the decreased CB1R surface expression resulting from SGIP1 knockdown is due to increased endocytosis, we again transfected DIV9 hippocampal neurons with EGFP–CB1R^WT^ and either a scrambled shRNA (SCR29) or an SGIP1-targeting shRNA. At 5 days after transfection, neurons were treated with leupeptin to block internalised receptor degradation. Surface-expressed EGFP–CB1R^WT^ was then ‘pulse’ labelled with anti-GFP antibody and ‘chased’ after labelling for 1 h in 5 µM 2-arachidonoylglycerol (2-AG) to induce endocytosis. Any remaining surface anti-GFP antibody was then removed by acid washing, allowing the extent of agonist-induced CB1R internalisation to be measured (Fig. 4A). SGIP1 knockdown significantly increased accumulation of endocytosed CB1R in the soma, but not in axons or dendrites (Fig. 4B; Fig. S4), suggesting that upon agonist-induced internalisation, receptors undergo retrograde trafficking to the soma (Roney et al., 2022), where they accrue because degradation is blocked. These data are therefore consistent with a role for SGIP1 in stabilising CB1R expression at the cell surface.

### SGIP1 knockdown reduces the surface:total ratio of endogenous CB1R

**Fig. 5.**
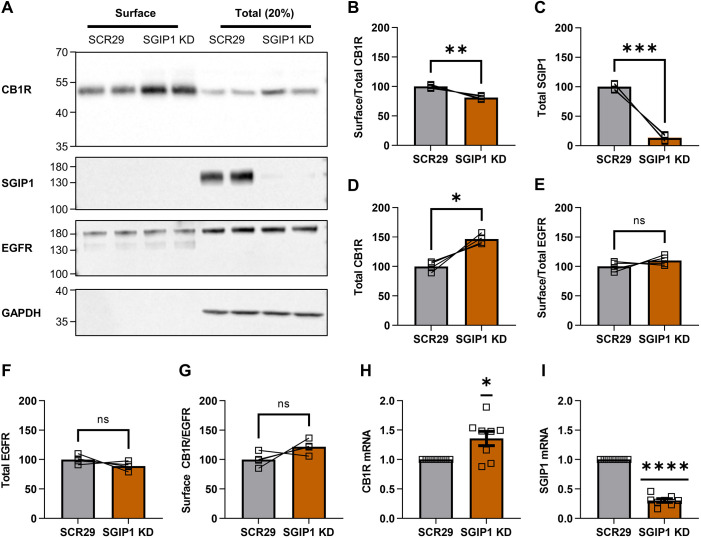
**SGIP1 knockdown decreases the surface**:**total ratio of endogenous CB1R.** (A) Representative immunoblots showing surface (left) and total (right; 20%) levels of endogenous CB1R in DIV14 cortical neurons transduced with SCR29 or SGIP1-targeting shRNA (SGIP1 KD). Blots were probed with anti-SGIP1 to determine efficiency of SGIP1 knockdown. Only experiments with >80% efficiency of SGIP1 knockdown were analysed. EGFR was included as a control surface protein and GAPDH was included as a loading control. The two lanes for each condition represent duplicate experiments from the same neuronal culture preparation. Positions of molecular mass markers are indicated in kDa. (B–G) Quantification of data represented in A, expressed as percentage of the SCR29 control. Paired two-tailed *t*-tests (*t* statistics are shown with degrees of freedom indicated in parentheses); *n*=4 independent experiments (values for each independent experiment are the average of duplicates). (B) Surface:total ratio of CB1R is significantly reduced with SGIP1 KD compared to SCR29 control [SCR29 versus SGIP1 KD: mean±s.e.m., 100.00±1.48 versus 81.23±1.48; *t*(3)=6.364, **P=0.0079]. (C) SGIP1 levels are knocked down by ∼87% with SGIP1 KD compared to SCR29 control [SCR29 versus SGIP1 KD: mean±s.e.m., 100.00±2.82 versus 13.10±2.82; *t*(3)=15.42, ***P=0.0006]. (D) Total levels of CB1R are significantly increased with SGIP1 KD compared to SCR29 control [SCR29 versus SGIP1 KD: mean±s.e.m., 100.00±4.22 versus 146.40±4.22; *t*(3)=5.493, *P=0.0119]. (E) SGIP1 KD has no significant effect on EGFR surface:total levels [SCR29 versus SGIP1 KD: mean±s.e.m., 100.00±4.18 versus 110.10±4.18; *t*(3)=1.210, *P*=0.138]. (F) SGIP1 KD has no significant effect on EGFR total levels [SCR29 versus SGIP1 KD: mean±s.e.m., 100.00±3.94 versus 81.23±3.94; *t*(3)=1.406, *P*=0.094]. (G) There is no significant difference in absolute levels of surface CB1R upon SGIP1 KD when normalised to surface EGFR [SCR29 versus SGIP1 KD: mean±s.e.m., 100.00±6.28 versus 121.5±6.28; *t*(3)=1.714, *P*=0.1851]. (H,I) RT-qPCR of DIV14 and DIV15 cortical neurons transduced with SCR29 or SGIP1 KD lentivirus. Cycle threshold (Ct) values for the gene of interest were normalised to those for *Gapdh* (ΔCt) and presented as fold change compared to SCR29 control (2^−ΔΔCt^). In H,I, two-tailed one-sample *t*-tests (theoretical mean=1; *t* statistics are shown with degrees of freedom indicated in parentheses); *n*=8 independent experiments. (H) Knockdown of SGIP1 increases CB1R mRNA levels [SGIP1 KD: mean±s.e.m., 1.367±0.1206 versus theoretical mean=1.000; *t*(7)=2.963, *P=0.0210]. (I) SGIP1 KD lentivirus reduced SGIP1 transcript levels by ∼70% (SGIP1 KD: mean±s.e.m., 0.3027±0.03127 versus theoretical mean=1.000; t(7)=22.30, ****P<0.0001). ns, not significant.

To determine how SGIP1 affects surface expression of endogenous CB1R, we transduced DIV7 or DIV8 primary cortical neurons with lentivirus expressing either a scrambled shRNA control (SCR29) or SGIP1-targeting shRNA. Surface and total levels of endogenous CB1R were examined at DIV14 or DIV15 by surface biotinylation followed by streptavidin pulldown and western blotting (Fig. 5A). SGIP1-targeting shRNA decreased SGIP1 levels to ∼15% of those in SCR29 shRNA control neurons and, consistent with our data using exogenously expressed CB1R, significantly decreased the proportion of endogenous CB1R expressed on the cell surface (Fig. 5B,C; Fig. S1). These results further support a role for SGIP1 in promoting CB1R surface expression.

To our surprise, however, in these experiments, total levels of endogenous CB1R were increased by SGIP1 knockdown compared to CB1R levels in the SCR29 control (Fig. 5D). In contrast, neither surface (Fig. 5E) nor total (Fig. 5F) levels of another surface expressed receptor, epidermal growth factor receptor (EGFR), were affected by SGIP1 knockdown. These results suggest that SGIP1 knockdown has selective effects and does not evoke global changes in membrane protein levels or surface expression. Moreover, normalisation of surface CB1R levels to surface EGFR levels showed that the increase in total CB1R protein levels restores absolute surface CB1R levels in SGIP1-knockdown cells (Fig. 5G).

To assess whether this increase in total CB1R protein levels was due to increased transcription, we used RT-qPCR to analyse transcript levels of CB1R in DIV14 and DIV15 cortical neurons transduced with SCR29 or SGIP1-targeting shRNA (Fig. 5H,I; Fig. S5). Interestingly, the relative mRNA level of CB1R was significantly increased when SGIP1 was knocked down. From these data we hypothesise that increased CB1R transcription may constitute a homeostatic feedback mechanism triggered in response to the reduced CB1R surface expression resulting from SGIP1 knockdown.

### SGIP1 knockdown impairs CB1R-mediated inhibition of intracellular Ca^2+^ influx

We next investigated how ablation of SGIP1 affects presynaptic CB1R signalling. CB1R and SGIP1 co-expression in cell lines alters CB1R-mediated, pertussis toxin-sensitive ERK1/2 phosphorylation as well as recruitment of β-arrestin2 and GRK3 (Gazdarica et al., 2022; Hajkova et al., 2016), whereas G_i/o_ protein activation and G_q_ protein-mediated intracellular Ca^2+^ mobilisation is unaffected (Hajkova et al., 2016). In autaptic hippocampal neurons, loss of SGIP1 modulates depolarisation-induced suppression of excitation and 2-AG-mediated inhibition of excitatory postsynaptic currents but does not affect desensitisation (Straiker et al., 2023).

**Fig. 6.**
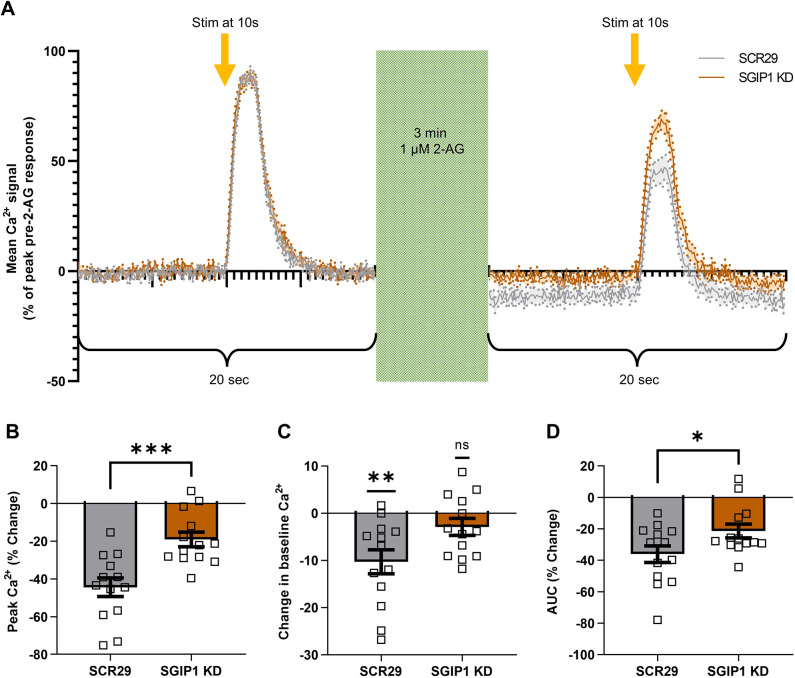
**SGIP1 knockdown decreases CB1R-mediated inhibition of Ca^2+^ influx.** (A) Average traces of presynaptic Ca^2+^ responses (measured using SyGCaMP3) to 20 APs at 20 Hz before (left) and after (right) 2-AG incubation (1 μM, 3 min). Data are normalised to basal levels (set to 0; calculated as average mean fluorescence during seconds 8–9) and expressed as a percentage of the peak response before 2-AG incubation. Solid line marks the mean; shading and dotted line indicate the s.e.m.; *n*=13 fields of view from eight independent neuronal cultures. Stim, field stimulation. (B–D) Quantification of data represented in A. (B) SGIP1-targeting shRNA (SGIP1 KD) significantly reduces the drop in peak Ca^2+^ levels after 2-AG application compared to the SCR29 control [SCR29 versus SGIP1 KD: mean±s.e.m., −45.45±4.92 versus −19.07±3.84; *n*=13 versus *n*=13; unpaired two-tailed *t*-test, *t*(24)=4.066, ***P=0.0004]. *n*=13 fields of view from eight independent neuronal cultures. (C) Application of 2-AG significantly reduces the baseline level of Ca^2+^ of SCR29-transfected control neurons but not of SGIP1 KD neurons. Difference between average mean fluorescence during seconds 8–9 before and after 2-AG application. SCR29 versus 0: mean±s.e.m., −10.29±2.55; *n*=13; *t*(12)=0.0017, **P=0.0017. SGIP1 KD versus 0: mean±s.e.m., −2.89±1.80; *n*=13; *t*(12)=1.607, *P*=0.134. Two-tailed one sample *t*-tests with theoretical mean=0 were used. *n*=13 fields of view from eight independent neuronal cultures. (D) SGIP1 KD significantly reduces the area under curve (AUC) of the Ca^2+^ signal after 2-AG application compared to SCR29 control [SCR29 versus SGIP1 KD: mean±s.e.m., −36.06±5.30 versus −21.36±4.38; *n*=13 versus *n*=13; unpaired two-tailed *t*-test, *t*(24)=2.139, *P=0.043). *n*=13 fields of view from eight independent neuronal cultures. The *t* statistics are shown with degrees of freedom indicated in parentheses. ns, not significant.

We transfected DIV8 and DIV9 primary hippocampal neurons with the presynaptically localised Ca^2+^ indicator synaptophysin–GCaMP3 (SyGCaMP3) (Girach et al., 2013) and either SCR29 or SGIP1-targeting shRNA, and assayed CB1R function at DIV14 or DIV15. Neurons were subjected to field stimulation (50 V, 1 ms pulses) to evoke 20 action potentials (APs) at 20 Hz. They were then perfused with the CB1R agonist 2-AG (1 μM) for 3 min and then restimulated to compare the Ca^2+^ signal before and after 2-AG incubation (Fig. 6A).

As expected, in control neurons the peak Ca^2+^ signal decreased after 2-AG incubation by ∼45% (Fig. 6B), which is consistent with the presynaptic inhibitory action of CB1R signalling (Pan et al., 1996; Twitchell et al., 1997). The magnitude of this decrease was markedly reduced in SGIP1-knockdown neurons (Fig. 6B). Furthermore, 2-AG incubation significantly decreased the baseline Ca^2+^ signal compared to that before 2-AG incubation in control cells, but this did not occur after SGIP1 knockdown (Fig. 6C). Lastly, the reduction in the area under the curve of the Ca^2+^ response was significantly less in SGIP1-knockdown cells compared to control cells (Fig. 6D). Taken together, these data suggest that SGIP1 knockdown suppresses the endocannabinoid-mediated reduction in Ca^2+^ influx, indicative of reduced presynaptic CB1R signalling in the absence of SGIP1.

## DISCUSSION

The context of this study was that the amphipathic α-helical H9 domain in the intracellular C-terminal region of CB1R contributes to the polarised presynaptic surface expression of CB1R (Fletcher-Jones et al., 2019). SGIP1 binds to CB1R, increasing its surface expression and modulating its signalling in HEK293 cells (Gazdarica et al., 2022; Hajkova et al., 2016). The distributions of SGIP1 and CB1R overlap in mouse brain (Lein et al., 2007), and the two proteins co-localise at the presynapse (Hajkova et al., 2016). We therefore hypothesised that SGIP1 might interact with the H9 domain to modulate synaptic CB1R availability at the presynaptic membrane.

We show that SGIP1 binds to the CB1R H9 domain to promote axonal surface expression. A FLAG-tagged isoform of SGIP1, which we refer to as SGIP1β, co-immunoprecipitates with ctCB1R^WT^ but not with ctCB1R^ΔH9^ in HEK293T cells (Fig. 1A–C). Moreover, expression of SGIP1β increases CB1R axonal plasma membrane localisation (Fig. 2), whereas knockdown of SGIP1 reduces axonal surface levels (Fig. 3) and increases accumulation of CB1R in somatic endolysosomes after agonist stimulation (Fig. 4). Importantly, neither exogenous expression (Fig. 2) nor knockdown (Fig. 3) of SGIP1 affects CB1R^ΔH9^ surface expression, which is consistent with SGIP1 mediating its effect through interaction with the H9 domain of CB1R.

SGIP1 knockdown both reduces the surface:total ratio of endogenous CB1R (Fig. 5A–C) and modulates downstream CB1R signalling (Fig. 6). Under control conditions, CB1R activation inhibits voltage-gated Ca^2+^ channels via G_i_ βγ-subunit mobilisation (Pan et al., 1996; Twitchell et al., 1997), but the extent of this inhibition is significantly reduced following SGIP1 knockdown (Fig. 6). Taken together, these data indicate that SGIP1 promotes axonal surface localisation of CB1R through interaction with the H9 domain.

Intriguingly, although SGIP1 knockdown markedly reduced the proportion of endogenous CB1R expressed at the surface, indicating a CB1R trafficking defect under these conditions (Fig. 5B), the absolute amount of CB1R expressed on the surface was unchanged compared to that observed for control cells (Fig. 5G), as a result of a transcription-dependent increase in total levels of CB1R (Fig. 5H,I). We hypothesise that this represents a homeostatic response mechanism to counter the decreased CB1R surface expression resulting from SGIP1 knockdown. Nonetheless, these findings raise the question of how SGIP1 knockdown reduces CB1R-dependent inhibition of Ca^2+^ influx if absolute CB1R surface levels are restored. However, it is important to note that our experiments using surface biotinylation assess whole-cell surface expression of endogenous CB1R and therefore would not detect differences in the distribution of CB1R on the neuronal surface, or its clustering and enrichment at presynaptic sites, which are likely crucial to CB1R signalling. Furthermore, our endocytosis assay (Fig. 4) also indicates that residency time of CB1R at the plasma membrane is reduced by SGIP1 knockdown, suggesting that although surface levels might be restored under basal conditions, in response to an agonist, activated CB1R receptors are quickly removed from the plasma membrane. Finally, work by the Blahos and Mackie groups suggests that the presence or absence of SGIP1 can have an allosteric effect on CB1R signalling (Durydivka et al., 2023; Gazdarica et al., 2022; Hajkova et al., 2016) and might therefore affect signalling in the absence of overt changes in CB1R surface levels. Further studies will be required to determine how SGIP1 loss affects CB1R localisation in specific subdomains of the axon, and exactly how SGIP1 supports CB1R-dependent Ca^2+^ signalling at the presynapse.

It is also important to note that as SGIP1 is a presynaptically enriched cargo adaptor, SGIP1 knockdown might have effects on the presynapse beyond CB1R. Therefore, although we show reduced endocannabinoid-mediated Ca^2+^ influx in response to stimulation, this study does not exclude the possibility that this difference is in fact due to altered Ca^2+^ handling, such as the loss of voltage-gated Ca^2+^ channels themselves. More studies will be required to test this possibility directly.

SGIP1 is a member of the muniscin family of cargo adaptors due to its similarity with FCHo1 and FCHo2 proteins (collectively termed FCho1/2), and it interacts with endophilin (Trevaskis et al., 2005) the AP-2 adaptor complex (Hollopeter et al., 2014) intersectin 1 (ITSN1), amphiphysin 1 (Dergai et al., 2010) and Eps15 (Uezu et al., 2007). Since SGIP1 is a component of the clathrin-mediated endocytosis complex, a key question is why does SGIP1 overexpression enhance, and SGIP1 knockdown reduce*,* CB1R surface expression?

We speculate that key to untangling this conundrum is the observation that the actions of SGIP1 are highly isoform dependent (Lee et al., 2021). Both SGIP1 and FCHo1/2 proteins contain an N-terminal MP domain, an AP-2 activator domain, a PRD and a C-terminal µ homology domain (Dergai et al., 2010; Hollopeter et al., 2014) (Fig. 1A). However, the MP domain of FCHo1/2 is an F-BAR domain that deforms the plasma membrane to facilitate clathrin-coated pit formation, whereas the corresponding region of SGIP1 has no F-BAR sequence similarity.

Nonetheless, a recent report has identified a 28-residue, positively charged sequence present in the MP domain that is necessary for homo-oligomerisation of SGIP1 and membrane tubulation (Lee et al., 2021). Of the 32 different predicted rat transcript variants available on the NCBI database, 13 contain the membrane-tubulating sequence in its entirety. Importantly, SGIP1α contains this sequence, whereas SGIP1 and SGIP1β do not, and previous experiments indicating that SGIP1 and CB1R co-expression in HEK293 cells increases CB1R surface levels were performed using the non-membrane-tubulating form of SGIP1 (Hajkova et al., 2016). Consistent with these findings, our data show that overexpression of SGIP1β, which does not contain the tubulating sequence, increases CB1R surface levels in axons (Fig. 2).

We note, however, that different SGIP1 isoforms might be selectively recruited to different cargo to mediate opposing effects. Differences between the SGIP1 isoforms are in the homo-oligomerisation and membrane-tubulating sequences, whereas the C-terminal 99-residue domain of SGIP1 that interacts with CB1R is present in all three SGIP1 isoforms. One possibility could be that SGIP1 and SGIP1β act as endogenous ‘dominant negatives’ to SGIP1α and FCHo1/2 proteins, preventing them from binding cargo by taking up the binding site. However, further work will be required examine this possibility directly. In conclusion, our findings indicate that SGIP1 promotes CB1R surface expression via interaction with the H9 domain.

## MATERIALS AND METHODS

### Plasmids and reagents

To N-terminally tag rat CB1R, the first 25 N-terminal amino acids were removed and an exogenous signal peptide corresponding to interleukin-2 (SP^Il2^) was added before the tags, as previously characterised (Fletcher-Jones et al., 2019; McDonald et al., 2007a). The CB1R^ΔH9^ sequence lacked T440–V460 and was created by site-directed mutagenesis (Fletcher-Jones et al., 2019). The ctCB1R^WT^ and ctCB1^ΔH9^ sequences consisted of R401–L473 with or without the H9 domain and were created by standard PCR and ligation cloning techniques. The SGIP1β sequence was isolated from a cDNA library derived from DIV21 rat primary cortical neurons. Briefly, RNA from DIV21 rat primary cortical neurons was extracted using the RNeasy mini kit (QIAGEN) then 1 μg of RNA was converted into cDNA with the RevertAid First Strand cDNA synthesis kit (Thermo Fisher Scientific) using the provided oligo(dT)_18_ primer. SGIP1 was amplified by PCR using the KOD Hot Start DNA Polymerase Kit (Sigma-Aldrich) and the following primers: forward, 5′-CACGGTACCGAAGGACTGAAAAAACGTACAAGA-3′; reverse: 5′-GTGGGATCCTTAGTTATCTGCCAAGTATTTTCCTGCAGC-3′. The non-targeting 29-mer shRNA (SCR29) sequence was 5′-GCACTACCAGAGCTAACTCAGATAGTACT-3′ (Origene). The SGIP1 shRNA target sequence was 5′-CCAATACCAAGGAATTCTGGGTAAA-3′ (Uezu et al., 2007).

For overexpression of EGFP, EGFP–ctCB1R^WT^ and EGFP–ctCB1R^ΔH9^ (Fig. 1), the pEGFP-C2 (Clontech) vector was used. For overexpression of FLAG–SGIP1β (Fig. 1; Fig. S2), pcDNA3.1 (Invitrogen) with an N-terminal FLAG tag (DYKDDDDK) was used. For double overexpression of the SBP tag (DEKTTGWRGGHVVEGLAGELEQLRARLEHHPQGQREP) or SBP–SGIP1β with either EGFP–CB1R^WT^ or EGFP–CB1R^ΔH9^ (Fig. 2), the pXlg3-PX-GFP-WPRE vector (Wilkinson et al., 2022) was used to express SBP tag sequences from a CMV promoter and SP^Il2^–EGFP–CB1R sequences from an Sffv promoter. For experiments combining shRNA treatments with overexpression of CB1R sequences (Figs 3,4), the pXlg3-PX-GFP-WPRE vector was used to express either SCR29 or the SGIP1-targeting shRNA from an H1 promoter, and either SP^Il2^–EGFP–CB1R^WT^ or SP^Il2^–EGFP–CB1R^ΔH9^ from an Sffv promoter. For experiments combining shRNA treatments with overexpression of mCherry (Fig. 5; Fig. S1), pSUPER.neo (Oligoengine) or pXlg3 was used to express SCR29 or the SGIP1-targeting shRNA from an H1 promoter, and mCherry from an Sffv promoter. For SyGCaMP3 assays (Fig. 6), pXlg3 was used to express either SCR29 or the SGIP1-targeting shRNA from an H1 promoter and SyGCaMP3 (Girach et al., 2013) from an Sffv promoter. For lentivirus production, lentiviral helper vectors p8.91 (Addgene #12263; Wilkinson et al., 2022) and pMD2.G (Addgene #12259; Wilkinson et al., 2022) were used. All plasmids are available upon request from the corresponding authors.

Details of primary antibodies are provided in Table S1. Primary antibodies were used following suppliers' recommended protocols and validation profiles. Details of secondary antibodies are provided in Table S2. All fluorescently labelled secondary antibodies for immunocytochemistry were used at 1:400, and all HRP-conjugated secondary antibodies for western blotting were used at 1:10,000.

### Cell culture and transfection

The hippocampus and cortex were dissected from Han Wistar rats at embryonic day (E)17 and dissociated according to standard protocols (Nair et al., 2021). A total of 200,000–350,000 dissociated hippocampal neurons were plated onto 25 mm glass coverslips pre-coated with poly-D-lysine (PDL; Sigma) in plating medium [Neurobasal (Gibco) supplemented with 5% horse serum (Sigma), 2 mM GlutaMAX (Gibco) and 1× GS21 (GlobalStem)]. A total of 500,000 cortical neurons were plated per well of a 6-well plate pre-coated with PDL. The next day, the plating medium was removed and replaced with feeding medium (Neurobasal supplemented with 1.2 mM GlutaMAX and 1× GS21). When GS21 became unavailable, dissociated hippocampal neurons were both plated and maintained in B27 Plus medium [Neurobasal Plus supplemented with 1× B27 Plus (Gibco)].

Primary hippocampal neurons at DIV9 (for knockdown experiments) or DIV12 (for overexpression experiments) were transfected with 1–2 μg plasmid DNA and 3–5 μl Lipofectamine 2000 (Invitrogen) according to manufacturer's instructions, with minor modifications. For overexpression experiments, cells were left for 2–3 days, whereas for knockdown experiments, cells were left for 5 days to ensure efficient knockdown. All experiments were carried out at DIV14 or DIV15. Cortical neurons were transduced with lentivirus at DIV7 or DIV8 and assayed at DIV14 or DIV15. To limit glial growth, antimitotics [5-fluoro-2'-deoxyuridine (Merck) and uridine (Merck); final concentration 0.4 μM each] were added at DIV7 or DIV8 to cortical cultures.

Animal care and procedures were carried out in accordance with UK Animals (Scientific Procedures) Act 1986 and University of Bristol and ARRIVE guidelines. All experimental protocols were approved by the University of Bristol Animal Welfare and Ethics Review Body (AWERB; approval numbers UB/18/004 and UIN/23/069) panel and the Biological and Genetic Modification Safety Committee (BGMSC).

HEK293T cells (EACC) were passaged and maintained in glutamine-containing DMEM (Gibco) supplemented with 10% FBS (Merck) and 1% penicillin-streptomycin (Merck). HEK293T cells were treated with ciprofloxacin (10 μg ml^−1^; Merck) regularly to prevent mycoplasma contamination. HEK293T cells were passaged a maximum of 20 times before returning to frozen second passage (P2) aliquots and were regularly screened for mycoplasma contamination (Eurofins mycoplasma testing service). HEK293T cells were transfected 24 h after plating in 6 cm dishes. A total of 5 μg plamid DNA and 7.5 μl Lipofectamine 2000 was added to 500 μl plain DMEM. The transfection mix was briefly vortexed, centrifuged and incubated at room temperature for 20 min, then added to the dish. The cells were then returned to the incubator for 48–72 hours.

### GFP-Trap

GFP was immunoprecipitated with GFP-Trap (Chromotek) according to the manufacturer's instructions, with minor modifications. Samples were kept at 4°C throughout. HEK293T cells were lysed in lysis buffer [50 mM Tris-HCl pH 7.4, 150 mM NaCl, 0.5% Triton X-100, 1×cOmplete protease inhibitors (1 tablet in 40 ml; Merck)], sonicated, incubated for 20 min, and clarified by centrifugation at 16,000 ***g*** for 20 min. A proportion of the lysate was kept aside (‘input’), and the rest was incubated with GFP-Trap beads on a rotating wheel for 1 h. The beads were pelleted for 2 min at 1500 ***g*** and washed 3× in wash buffer (lysis buffer minus protease inhibitors). Then, 2× Laemmli sample buffer was added to the beads, and the inputs and the beads were boiled at 95°C for 5 min.

### Lentivirus production and transduction of cortical neurons

Lentivirus was produced in HEK293T cells following standard protocols (Wilkinson et al., 2022). HEK293T cells plated in 6- or 10-cm dishes were transfected using plain DMEM containing 2 μg ml^−1^ of the appropriate pXlg3 viral vector, 0.5 μg ml^−1^ pMD2.G, 1.5 μg ml^−1^ p8.91 and 12 μg ml^−1^ polyethylenimine (PEI; Sigma) in plain DMEM medium for 4 h. The transfection mix was removed and replaced with DMEM containing 10% FBS. After 48 h, the virus-containing medium was collected, centrifuged at 2800 ***g*** for 10 min, and passed through a 0.45 μm syringe filter to remove any remaining HEK293T cells. 500 μl of virus was added per well of a 6-well plate of DIV7 cortical neurons in duplicate and incubated for 7 days.

### Surface biotinylation and streptavidin pulldown

All solutions were pre-chilled to 4°C and steps were carried out on ice. DIV14 and DIV15 cortical neurons in 6-well plates transduced with lentivirus were cooled on ice to prevent endocytosis, then washed three times in ice-cold PBS. Surface proteins were biotinylated by incubation with 0.3 mg ml^−1^ EZ-link Sulfo-NHS-SS-Biotin (Thermo Fisher Scientific) dissolved in PBS for 10 min. Unreacted biotin was washed off with three washes in PBS and quenched with a 2 min incubation in 50 mM NH_4_Cl in PBS. Quenching solution was washed off with an additional three washes in PBS, and cells were lysed in 250 μl of lysis buffer (50 mM Tris-HCl pH 7.4, 150 mM NaCl, 1% CHAPS, 0.1% SDS, 10% glycerol, 1 mM EDTA, 1×cOmplete protease inhibitors), sonicated, incubated 20 min and clarified by centrifugation at 16,000 ***g*** for 20 min.

Biotinylated surface proteins were isolated using streptavidin-coated agarose beads (Merck). The beads were washed twice in lysis buffer by centrifugation at low speed (<1500 ***g***). 50 μl of clarified lysate was set aside (total), and 100 µl of clarified lysate was added to 30 µl of beads along with 500 µl of lysis buffer (surface). The beads were incubated on a rotating wheel at 4°C for 1.5 h, then washed in wash buffer (lysis buffer without protease inhibitors) three times, by pelleting the beads for 2 min at 1000 ***g*** and discarding the supernatant between washes. 2× Laemmli sample buffer was added to both the surface and total samples. The samples were vortexed, spun down and incubated overnight at room temperature (to prevent CB1R aggregation).

### Western blotting

Samples in Laemmli sample buffer were resolved on 10% acrylamide gels by SDS–PAGE, transferred onto methanol-activated PVDF membrane (Immobilon), and immunoblotted according to standard protocols. Briefly, membranes were blocked for 1 h at room temperature in 6% (w/v) non-fat milk powder in PBS with 1% Tween 20 (PBST), then incubated in primary antibody diluted in 6% milk-PBST overnight at 4°C. Following three 5-min washes in PBST, membranes were incubated in HRP-conjugated secondary antibody diluted in 6% milk-PBST for 1 h at room temperature, then washed an additional three times for 5 min each. Chemiluminescence was detected using a LI-COR Odyssey Fc and quantified using LI-COR Image Studio. For transparency, full, uncropped blots are available in Fig. S6.

### RT-qPCR

RNA was extracted from DIV14 and DIV15 cultured cortical cells transduced with lentivirus expressing a 29-mer non-targeting shRNA (SCR29) or SGIP1-targeting shRNA using the Qiagen RNeasy mini kit following manufacturer's instructions. RNA concentration was measured using a nanodrop, and 1 µg of RNA was converted to cDNA by reverse transcription using the RevertAid First Strand cDNA Synthesis Kit (Thermo Fisher Scientific) according to manufacturer's instructions.

qPCR was performed using PowerUp SYBR Green Master Mix (Thermo Fisher Scientific) mixed with 2 µl of each sample and gene-specific primers at 0.25 µM each and run on a qPCR machine with MxPro software and SYBR Green with a dissociation curve setup.

The following primers were used: CB1R forward, 5′-ACTCAGACTGCCTGCACAAG-3′; CB1R reverse, 5′-ACAGACATGGTCACCTTCGC-3′; SGIP1 forward, 5′-GTGAGGAAAAGTCCGAGGCG-3′; SGIP1 reverse, 5′-GAGTGTCATCCAGGGGCTTC-3′; GAPDH forward, 5′-AGTGCCAGCCTCGTCTCATA-3′; GAPDH reverse, 5′-GGTAACCAGGCGTCCGATAC-3′.

Unknown samples were run in triplicate, and no reverse transcription (noRT), no template (NTC) and SYBR negative (SYBR Neg; 10 µl SYBR master mix+10 µl H_2_O) controls were included with each qPCR.

Relative gene expression was quantified using the ΔΔCt method. Mean cycle threshold (Ct) values of the gene of interest were normalised first to *Gapdh* mean Ct values (ΔCt) and then to the SCR29 control (ΔΔCt). Fold change of gene expression was plotted as 2^−ΔΔCt^, and a two-tailed one-sample *t*-test was performed to determine whether the SGIP1-knockdown condition was significantly different from 1.

### Live surface staining

To measure surface expression, DIV14 cultured hippocampal neurons grown on 25 mm glass coverslips were incubated live in the appropriate antibody raised against an extracellular epitope. Briefly, cells were removed from the incubator and allowed to cool to room temperature for 5 min. Cells were then incubated in chicken anti-GFP antibody (1:1000) in 90 μl conditioned medium for 10 min at room temperature. The antibody mix was dotted onto parafilm, and the coverslips were incubated upside down to ensure even coating. Cells were washed three times in PBS to remove excess antibody and fixed.

### Endocytosis assay

To measure endocytosis, DIV14 cultured hippocampal neurons grown on 25 mm glass coverslips were pre-incubated for 3 h, and maintained throughout the assay, in 100 μg ml^−1^ leupeptin (Hello Bio) in conditioned medium to prevent subsequent degradation of endocytosed receptors. Surface receptors were labelled by incubation with chicken anti-GFP antibody (1:1000) for 10 min at room temperature and washed three times in osmolarity-matched HEPES Buffered Saline [HBS; 90–140 mM NaCl (adjusted according to the osmolarity of the culture medium), 5 mM KCl, 1.8 mM CaCl_2_, 0.8 mM MgCl_2_, 25 mM HEPES pH 7.4, 5 mM glucose] to remove excess antibody. Neurons were then returned to the incubator for 1 h in conditioned medium containing 5 μM 2-AG (Bio-Techne) to induce endocytosis. Remaining surface antibody was stripped with two quick washes in ice-cold PBS pH 2.5, and cells were fixed and stained.

### Fixation and fixed immunostaining

Cells were fixed in pre-warmed 4% paraformaldehyde and 5% sucrose in PBS for 12 min. Following three washes in PBS, residual paraformaldehyde was quenched with a wash in 100 mM glycine in PBS, and the cells were washed three more times in PBS.

Cells were blocked and permeabilised in 3% BSA in PBS with 0.1% Triton X-100 for 20 min. Cells were then incubated in secondary antibody to label surface or endocytosed receptors. Cells were then stained for total levels and ankyrin-G (ANK3; axon initial segment marker). Primary and secondary antibodies were diluted in 3% BSA in PBS. 90 μl of the antibody mix was dotted onto parafilm and the coverslips were incubated upside down for 1 h at room temperature. The cells were washed three times in PBS between incubations. Coverslips were dipped in distilled H_2_0 and mounted onto slides using Fluoromount G (Thermo Fisher Scientific) mounting media with or without DAPI.

### Fixed image acquisition and analysis

Images were acquired using a Leica SP8 confocal laser scanning microscope (Wolfson Bioimaging Facility, University of Bristol) with a 63×/1.40 oil objective. All settings were kept the same within experiments. To avoid bias, neurons were selected for data acquisition based only on their total staining, and surface staining was detected using a secondary antibody conjugated to a far-red fluorophore.

Fiji (ImageJ; https://fiji.sc/) was used to quantify fluorescence. Maximum-intensity projections of images were prepared, and regions of interest (ROIs) of approximately similar lengths were drawn around the axon and three dendrites based on the total staining channel only. Axons were defined as processes whose initial segment was positive for ankyrin-G, while dendrites were defined as processes negative for ankyrin-G. Surface fluorescence was normalised to total fluorescence for each ROI to control for differences in expression levels and expressed as a percentage of the axonal control condition. For endocytosis experiments, endocytosed fluorescence was normalised to total fluorescence for each ROI and expressed as a percentage of the soma control condition.

### SyGCaMP3 assay and analysis

SyGCaMP3 assays were performed as previously described (Girach et al., 2013). Hippocampal neurons grown on 25 mm coverslips were transfected at DIV8 or DIV9 with pXlg3-SCR29-SyGCaMP3 or pXlg3-SGIP1 KD-SyGCaMP3 and assayed at DIV14 or DIV15. Cells were assayed in HEPES Buffered Saline [HBS; 90–140 mM NaCl (adjusted according to the osmolarity of the culture medium), 5 mM KCl, 1.8 mM CaCl_2_, 0.8 mM MgCl_2_, 25 mM HEPES pH 7.4, 5 mM glucose] with 25 µM CNQX (Bio-Techne) and 50 µM D-AP5 (Bio-Techne) to prevent spontaneous firing. Time-lapse imaging of SyGCaMP3 was performed at 10 Hz with 2×2 binning on a Nikon Eclipse Ti-E C1 plus widefield microscope with a 40× objective, a CCD camera, a GFP filter cube and accommodating an electrical field stimulation setup. A 20 s imaging session was started. After 10 s of baseline recording, cells were electrically stimulated (50 V, 1 ms pulses) to evoke 20 APs at 20 Hz. Cells were then perfused with 1 μM 2-AG and incubated for 3 min, and the 20 s recording and stimulation were repeated.

For each field of view, the mean fluorescence of three to ten punctate ROIs was analysed, and non-responsive ROIs were discarded. The mean fluorescence of three background ROIs was also measured, which was subtracted for each timepoint for each ROI. Background subtracted values were then normalised to basal levels for each ROI (calculated as average mean fluorescence during 8–9 s). The average for each timepoint of all ROIs for a field of view was found and then plotted as a percentage of the peak mean fluorescence of the first stimulation. Three parameters were analysed: (1) change in peak signal after 2-AG treatment, (2) change in baseline (calculated as average during 8–9 s after 2-AG treatment) and (3) change in area under curve (AUC; total peak area was calculated using Graphpad Prism with the following parameters: baseline=mean of 8–9 s and 18–19 s, ignoring peaks less than 10% distance from minimum to maximum Y, ignoring any peaks defined by fewer than eight adjacent points; the AUC after 2-AG treatment was presented as a percentage of the AUC before 2-AG treatment).

### Statistics

All statistics were performed using GraphPad Prism (version 9). Outliers were removed using GraphPad Prism's ROUT method (Q=1%). To determine statistical significance between two groups, *t*-tests were used. For more than two groups, one- or two-way ANOVA with Tukey's or Sidak's post hoc tests were used to determine statistical significance, depending on the comparisons required.

For image analysis, *n* denotes the total number of neurons that were analysed, as is convention in the field (Coutts et al., 2001; Fletcher-Jones et al., 2019; Leterrier et al., 2006; McDonald et al., 2007a; Simon et al., 2013). However, the number of separate neuronal cultures prepared from litters of pups from separate dams is also noted for each experiment. For surface biotinylation experiments, *n* denotes the number of separate neuronal cultures, where each *n* is the average of two duplicate experiments. For all data, *P≤0.05, **P≤0.01, ***P≤0.001 and ****P≤0.0001. Data are presented as mean±s.e.m.

## Supplementary Material



10.1242/joces.261551_sup1 Supplementary information
