# Supplementary material for: SGIP1 binding to the α-helical H9 domain of cannabinoid receptor 1 promotes axonal surface expression
Source: J Cell Sci. 2024 Jun 12;137(11):jcs261551. doi: 10.1242/jcs.261551 (PMC11213518; doi:10.1242/jcs.261551)
Supplement: Supplementary information [file joces-137-261551-s1.pdf]

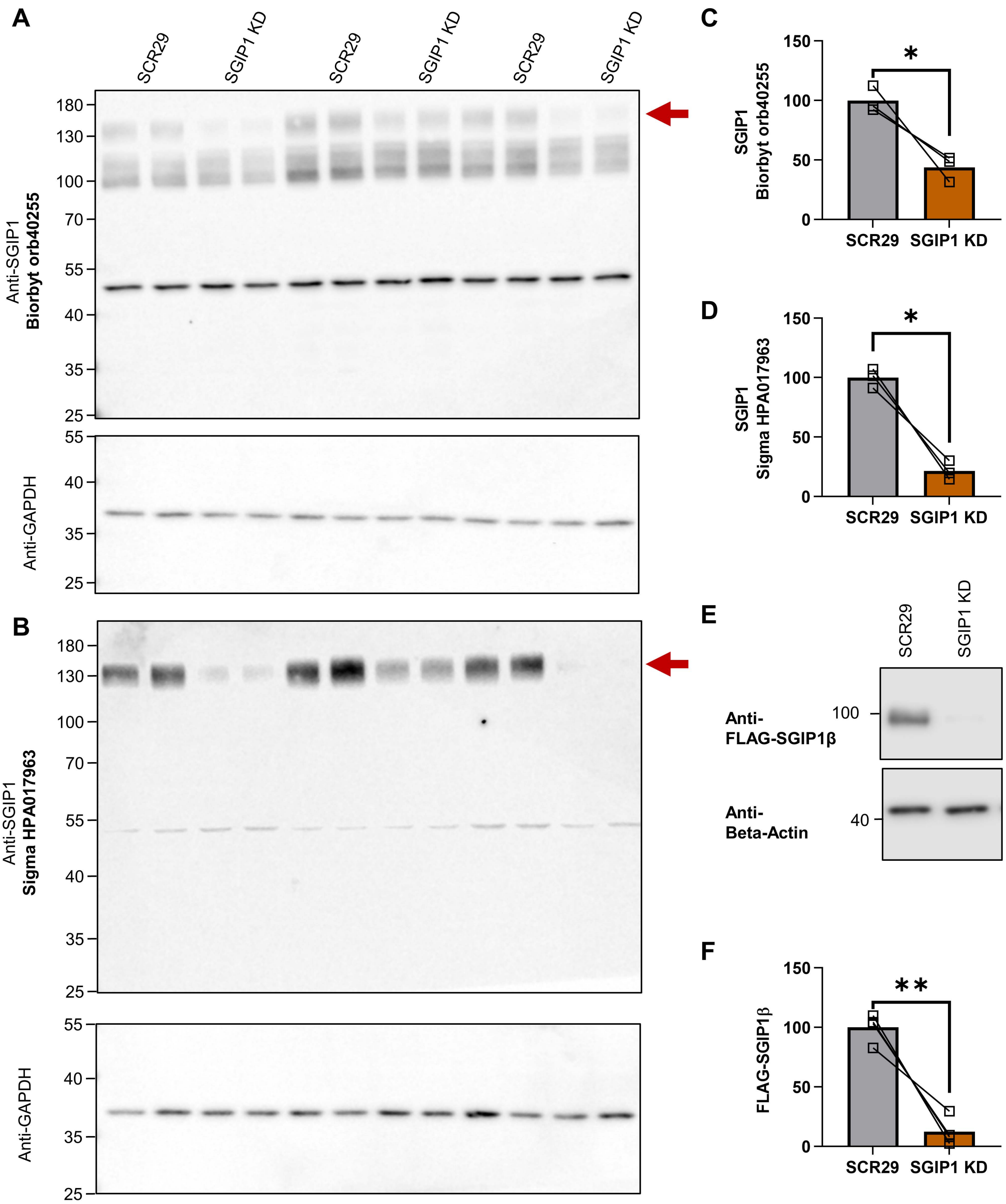

Journal of Cell Science • Supplementary information

Fig. S1. Validation of SGIP1 shRNA and antibodies

(**A-B**) Immunoblots of DIV14 cortical neuronal samples lentivirally transduced with SGIP1 shRNA or a 29mer scrambled control. Two different anti-SGIP1 antibodies were tested: (**A**) Biorbyt orb40255 and (**B**) Sigma HPA017963. Both antibodies recognised a band at around 130kDa (red arrow) that was reduced in intensity in SGIP1 shRNA samples compared to the 29mer scrambled control (**C-D**), suggesting this band is SGIP1 and/or SGIP1α. Biorbyt orb40255 also recognises a non-specific smear from around 100kDa to 120kDa and a band around 50kDa. Sigma HPA017963 also faintly recognised a band around 50kDa. This could represent a non-specific band or possibly a short isoform of SGIP1 or closely related protein that is insensitive to our shRNA.

SGIP1β was cloned out of our cortical neurons, suggesting that it may be one of the principal isoforms found in these cells, and FLAG-SGIP1β runs around 100kDa (see **E**). Sigma HPA017963 does not recognise a band at that molecular weight. However, this is likely because the antigen used to generate this antibody (residues K254-D345) almost completely overlaps with the deletion found in SGIP1β (N273-E438). The epitope used to generate Biorbyt orb40255 (M1-R30) is present in both SGIP1 and SGIP1β, but the non-specific smear from 100kDa to 120kDa likely obscures this band.

For clarity, all experiments in **Fig. 5** used the Sigma HPA017983 as it produced fewer non-specific bands, with the assumption that all isoforms of SGIP1 would be knocked down in a similar ratio.

Blots were stripped and reprobed for GAPDH as a loading control.

(**C**) Quantification of data represented in (**A**). Significant reduction in intensity of ~130kDa band recognised by Biorbyt orb40255 anti-SGIP1 antibody with SGIP1 knockdown (SCR29 vs. SGIP1 KD: mean ± s.e.m., 100.00 ± 6.295 vs. 43.83 ± 6.295, $t(2) = 4.462$, $^*p = 0.0467$). Paired two-tailed t-test. n = 3 independent experiments.

Journal of Cell Science • Supplementary information

(**D**) Quantification of data represented in (**B**). Significant reduction in intensity of ~130kDa band recognised by Sigma HPA017963 anti-SGIP1 antibody with SGIP1 knockdown (SCR29 vs. SGIP1 KD: mean ± s.e.m., 100.00 ± 4.613 vs. 21.46 ± 4.613, $t(2) = 8.513$, *$p = 0.0135$). Paired two-tailed t-test. n = 3 independent experiments.

(**E**) Representative immunoblots showing shRNA knockdown (KD) of overexpressed FLAG-SGIP1β and a beta-actin housekeeping control in HEK293T cells. FLAG-SGIP1β and a 25-mer SGIP1-targeting shRNA or a 29-mer non-targeting control (SCR29) shRNA were co-transfected into HEK293T cells and left for 3 days.

(**F**) Quantification of data represented in (**E**). The 25mer shRNA targeting SGIP1 knocked down overexpressed FLAG-SGIP1β by about 88% compared to a scrambled (non-targeting) 29mer control (SCR29) (SCR29 vs. SGIP1 KD: mean ± s.e.m., 100.00 ± 5.921 vs. 12.36 ± 5.921, $t(3) = 7.401$, **$p = 0.0051$). Paired two-tailed t-test. n = 4 independent experiments.

Journal of Cell Science • Supplementary information

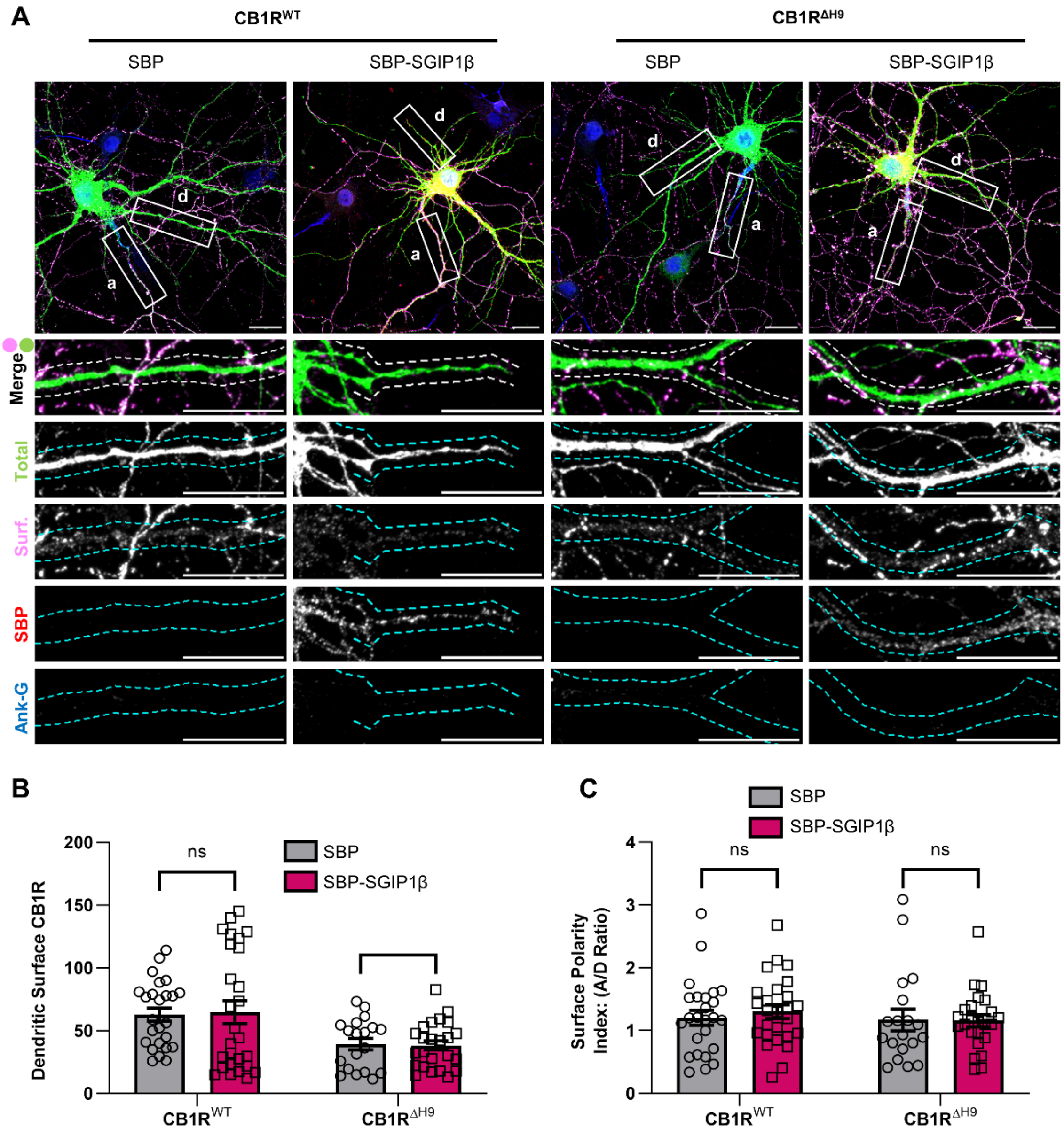

Journal of Cell Science • Supplementary information

Fig. S2. SGIP1 overexpression and dendrites

(**A**) Enlargements of dendritic ROIs of representative confocal images in **Fig. 2A**. Upper panels for each condition show whole cell field of view with white boxes indicating axonal (a) and dendritic (d) ROIs. Green = total; magenta = surface; red = SBP-SGIP1; blue = axon marker (Ankyrin-G). Merge: surface to total seen as white.

(**B**) Quantification of data represented in **Fig. 2A** and (**A**). Overexpression of SGIP1β isoform has no effect on dendritic surface levels of either CB1R^{WT} or CB1R$^{\Delta H9}$ (CB1R^{WT}/SBP vs. CB1R^{WT}/SBP-SGIP1β: mean ± s.e.m., 62.78 ± 5.25 vs. 64.71 ± 9.13; n = 25 vs. n = 27; $^{ns}p = 0.9699$. CB1R$^{\Delta H9}$/SBP vs. CB1R$^{\Delta H9}$/SBP-SGIP1β: mean ± s.e.m., 39.24 ± 4.63 vs. 38.30 ± 3.73; n = 19 vs. n = 24; $^{ns}p = 0.9940$). Surface fluorescence was normalised to total fluorescence and shown as a percentage of axonal CB1R^{WT}/SBP control (see **Fig. 2B**). Two-way ANOVA with Sidak's *post hoc* test; n = 19-27 neurons per condition from four independent neuronal cultures.

(**C**) Quantification of data represented in **Fig. 2A**. Overexpression of SGIP1β isoform has no effect on surface polarity (A/D) of either CB1R^{WT} or CB1R$^{\Delta H9}$ (CB1R^{WT}/SBP vs. CB1R^{WT}/SBP-SGIP1β: mean ± s.e.m., 1.203 ± 0.120 vs. 1.302 ± 0.108; n = 25 vs. n = 26; $^{ns}p = 0.7944$. CB1R$^{\Delta H9}$/SBP vs. CB1R$^{\Delta H9}$/SBP-SGIP1β: mean ± s.e.m., 1.170 ± 0.170 vs. 1.157 ± 0.096; n = 19 vs. n = 24; $^{ns}p = 0.9970$). Two-way ANOVA with Sidak's *post hoc* test; n = 19-26 neurons per condition from four independent neuronal cultures.

Journal of Cell Science • Supplementary information

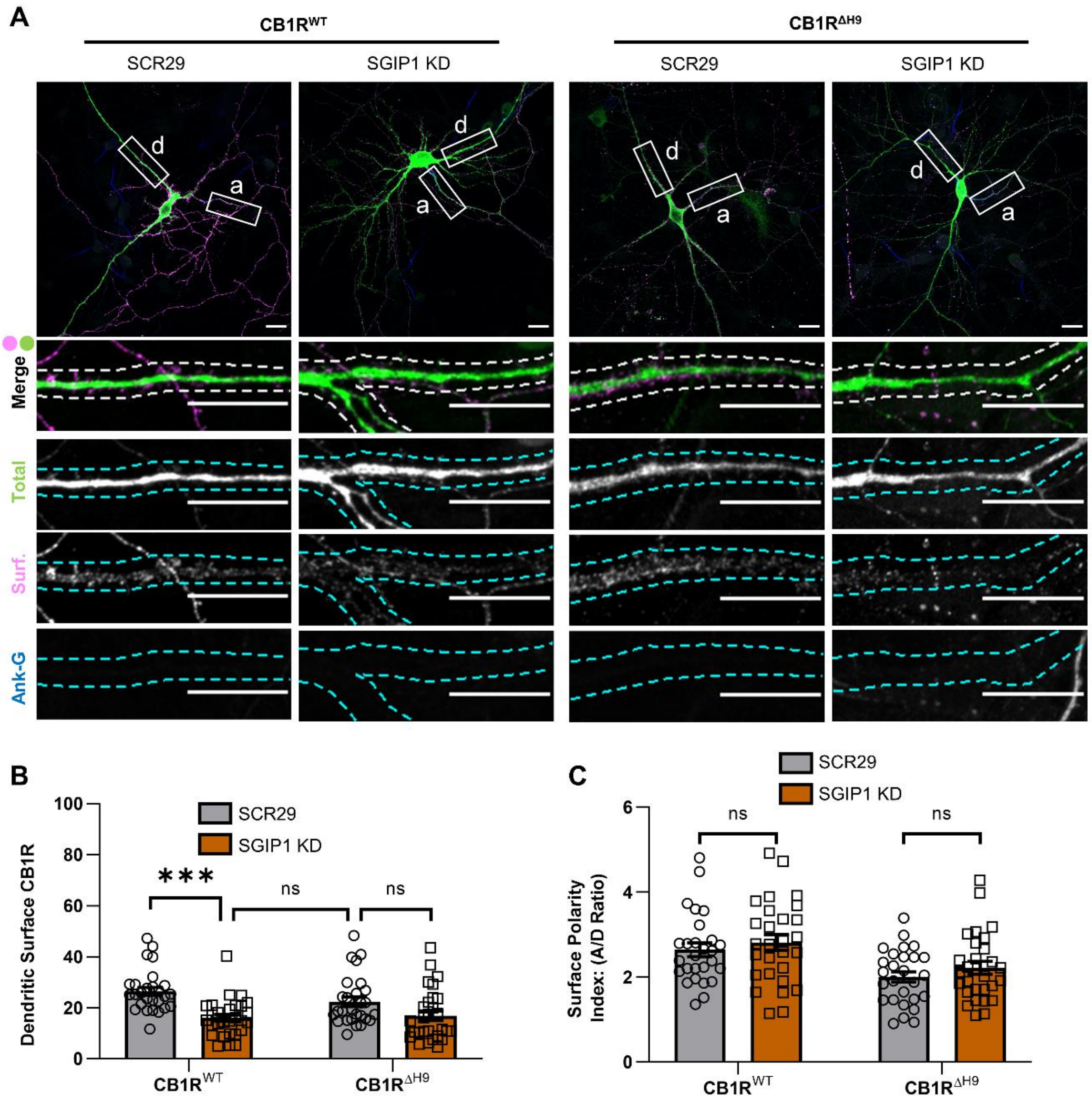

Fig. S3. SGIP1 KD and dendrites

(**A**) Enlargements of dendritic ROIs of representative confocal images in Fig. 3A. Upper panels for each condition show whole cell field of view with white boxes indicating axonal (a) and dendritic (d) ROIs. Green = total; magenta = surface; blue = axon marker (Ankyrin-G).

Journal of Cell Science • Supplementary information

(**B**) Quantification of data represented in **Fig. 3A**. Knockdown of SGIP1 reduces dendritic surface levels of $CB1R^{WT}$ ($CB1R^{WT}$/SCR29 vs. $CB1R^{WT}$/SGIP1 KD: mean ± s.e.m., 26.41 ± 1.60 vs. 16.04 ± 1.40; n = 27 vs. n = 28; ***p = 0.0002), but not $CB1R^{\Delta H9}$ ($CB1R^{\Delta H9}$/SCR29 vs. $CB1R^{\Delta H9}$/SGIP1 KD: mean ± s.e.m., 22.40 ± 1.88 vs. 16.88 ± 1.92; n = 27 vs. n = 28; $^{ns}p = 0.1390$), suggesting that an isoform other than SGIP1β may affect CB1R surface expression in dendrites. Surface fluorescence was normalised to total fluorescence and shown as a percentage of axonal $CB1R^{WT}$/ SCR29 (see **Fig. 3B**). Two-way ANOVA with Sidak's *post hoc* test. N = 27- 28 neurons from five independent neuronal cultures per condition.

(**C**) Quantification of data represented in **Fig. 3A**. Knockdown of SGIP1 has no effect on surface polarity (A/D) of either $CB1R^{WT}$ or $CB1R^{\Delta H9}$ ($CB1R^{WT}$/SCR29 vs. $CB1R^{WT}$/ SGIP1 KD: mean ± s.e.m., 2.638 ± 0.160 vs. 2.804 ± 0.185; n = 27 vs. n = 28; ^{ns}p = 0.7056. $CB1R^{\Delta H9}$/SCR29 vs. $CB1R^{\Delta H9}$/SGIP1 KD: mean ± s.e.m., 1.996 ± 0.126 vs. 2.211 ± 0.151; n = 27 vs. n = 28; $^{ns}p = 0.9664$). Two-way ANOVA with Sidak's *post hoc* test. N = 27-28 neurons from five independent neuronal cultures per condition.

Journal of Cell Science • Supplementary information

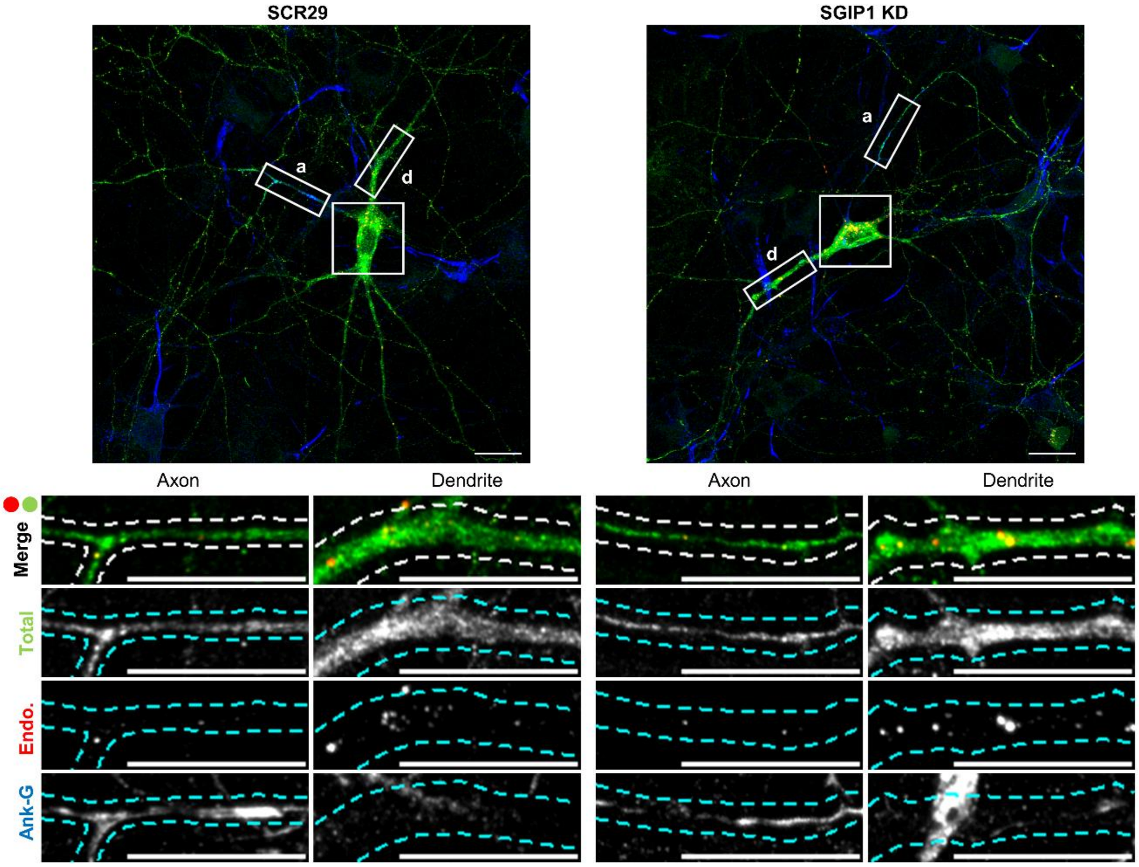

Fig. S4. CB1R Endocytosis in axons and dendrites

Enlargements of axonal and dendritic ROIs of representative confocal images in Fig. 4A quantified in Fig. 4B. Upper panels for each condition show whole cell field of view with white boxes indicating axonal (a) and dendritic (d) ROIs. Green = total; magenta = surface; blue = axon marker (Ankyrin-G). Green = total; red = endocytosed; blue = axon marker (Ankyrin-G). Merge: endocytosed to total seen as yellow.

CB1R primer set #1

For	5' - ACTCAGACTGCCTGCACAAG - 3'
Rev	5' - ACAGACATGGTCACCTTCGC - 3'

	Ct1	Ct2	Ct3	noRT
**SCR19**	19.31	19.05	19.03	29.67
**CB1R KD**	24.69	24.58	24.61	26.63

	Mean Ct	ΔCt	ΔΔCt	Fold diff
**SCR19**	19.13	2.62	0.00	1.00
**CB1R KD**	24.63	7.47	4.85	0.03

GAPDH

For	5' - AGTGCCAGCCTCGTCTCATA - 3'
Rev	5' - GGTAACCAGGCGTCCGATAC - 3'

	Ct1	Ct2	Ct3	noRT
**SCR19**	16.39	16.49	16.65	26.18
**CB1R KD**	16.96	17.53	16.99	23.25

	Mean Ct
**SCR19**	16.51
**CB1R KD**	17.16

**NTC CB1R**	32.58
**NTC GAPDH**	No Ct
**SYBR Neg. Cont.**	No Ct

Fig. 5. qPCR primers were validated against KD samples.

Specificity of CB1R qPCR primer set used in Fig. 5G was tested against DIV14 cortical neuronal samples lentivirally transduced with CB1R shRNA (target sequence from (Chen et al., 2012) or a 19mer scrambled control (target sequence from (Choy et al., 2014)). There was a 97% reduction in transcript levels in CB1R KD neurons compared to SCR19 control, suggesting that the primers used are specific for CB1R mRNA.

Journal of Cell Science • Supplementary information

Figure 1A

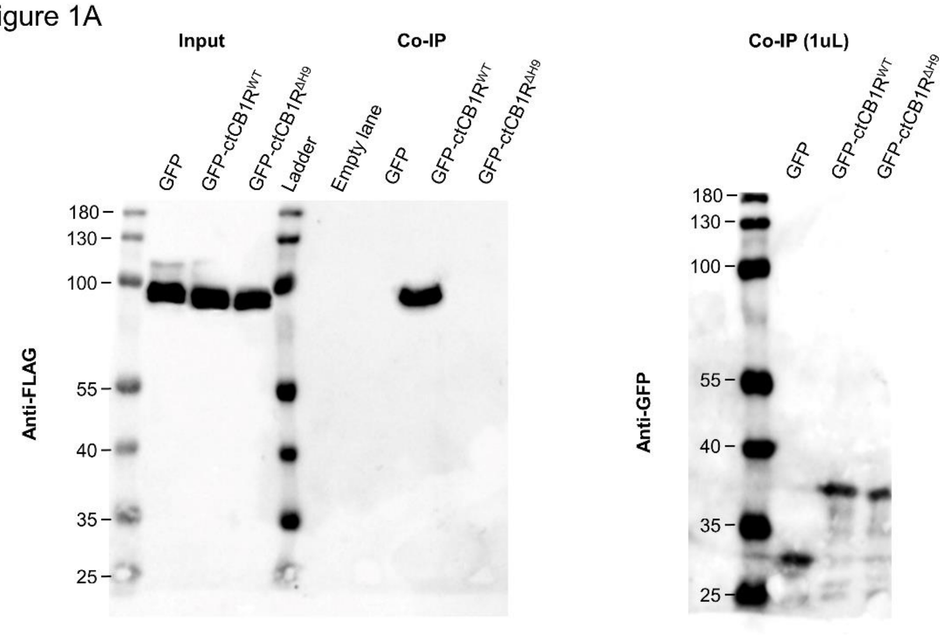

Figure 5A

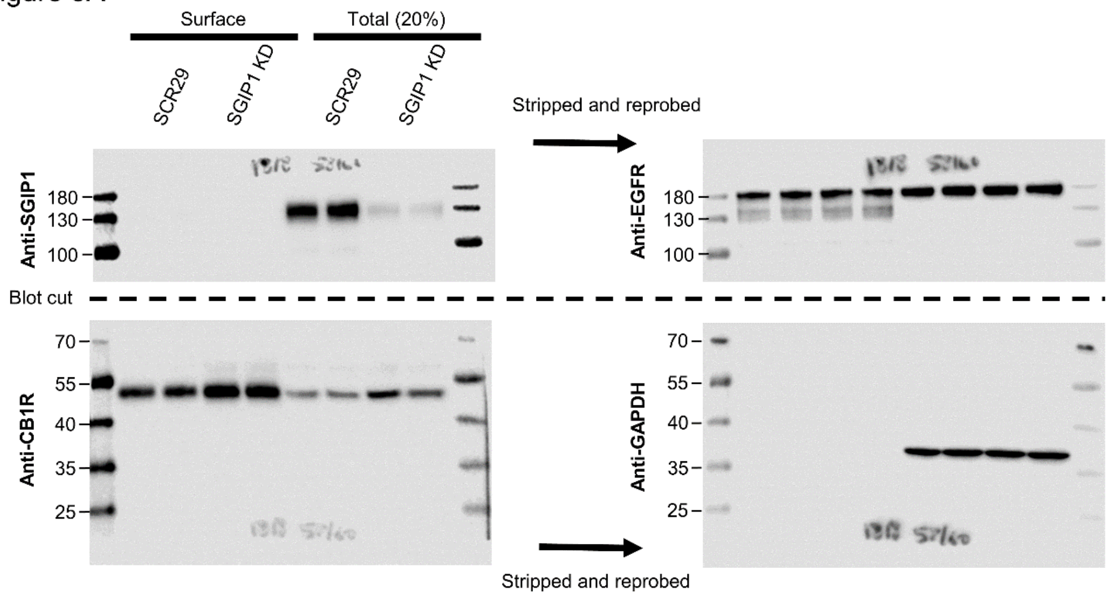

Journal of Cell Science • Supplementary information

Supplementary Figure 1 A-B

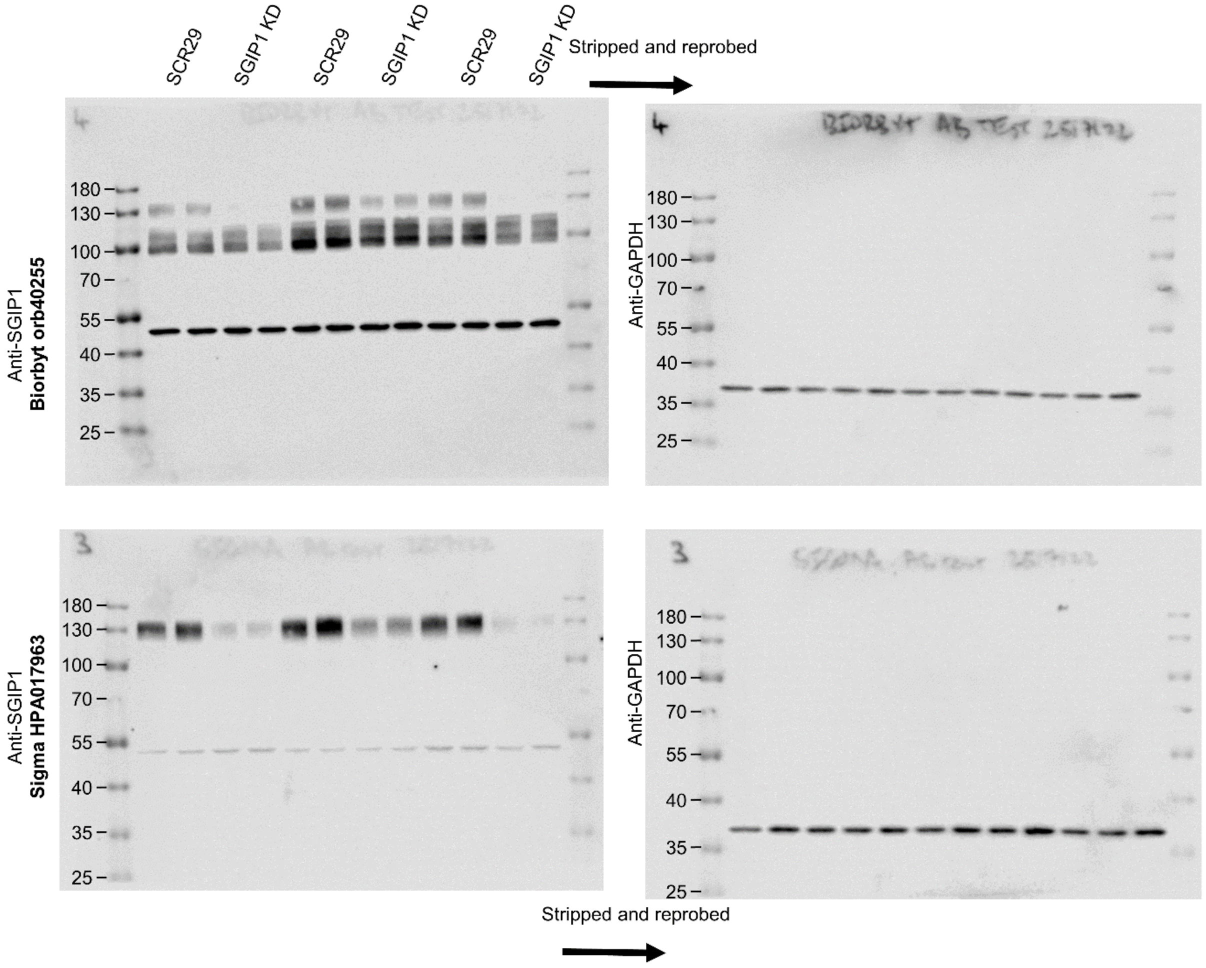

Supplementary Figure 1E

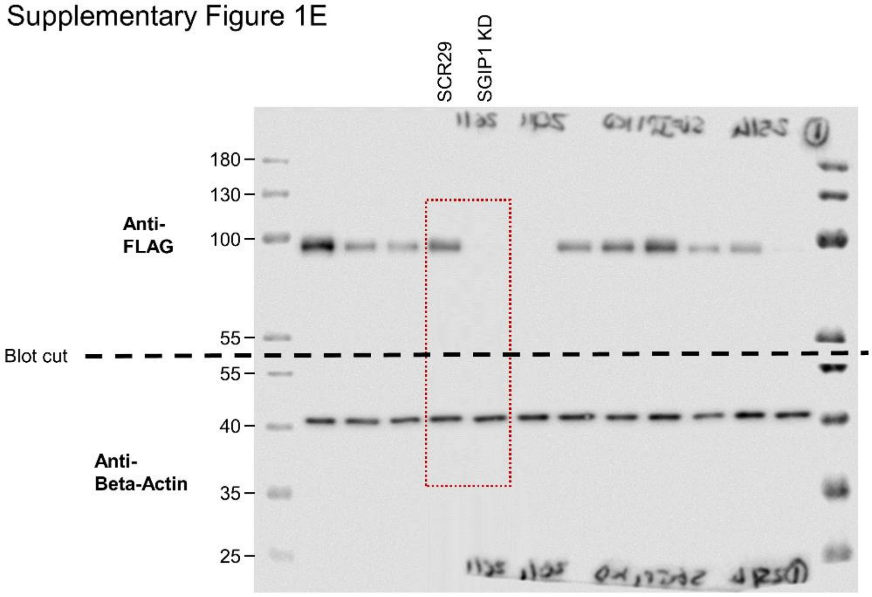

Fig. S6. Blot transparency

Journal of Cell Science • Supplementary information

Table S1. Primary antibodies.

Antibody	Clone	Source	Identifier	Concentration
anti-GFP (chicken)	Poly	Abcam	Cat# ab13970, RRID:AB_300798	ICC: 1:1,000
Anti-Ankyrin G (mouse)	N106/ 36	UC Davis/NIH NeuroMab Facility	Cat# N106/36, RRID:AB_2877524	ICC: 1:500
Anti-Ankyrin G (rabbit)	Poly	Synaptic Systems	Cat# 386 003, RRID:AB_2661876	ICC: 1:500
Anti-SBP-tag (mouse)	20	Millipore	Cat# MAB10764, RRID:AB_10631872	ICC: 1:500
Anti-FLAG (mouse)	M2	Sigma-Aldrich	Cat# F1804, RRID:AB_262044	WB: 1:2,000-1:10,000
Anti-GFP (rat)	3H9	ChromoTek	Cat# 3h9-100, RRID:AB_10773374	WB: 1:2,000-1:5,000
Anti-CB1R (rabbit)	Poly	Abcam	Cat# ab23703, RRID:AB_447623	WB: 1:250-1:1,000
Anti-SGIP1 (rabbit)	Poly	Sigma-Aldrich	Cat# HPA017963, RRID:AB_10964882	WB: 1:100
Anti-SGIP1 (rabbit)	Poly	Biorbyt	Cat# orb40255, RRID:AB_10997021	WB: 100
Anti-EGFR (rabbit)	EP38Y	Abcam	Cat# ab52894, RRID:AB_869579	WB: 1:1,000
Anti-GAPDH (mouse)	6C5	Abcam	Cat# ab8245, RRID:AB_2107448	WB: 1:10,000-1:40,000
Anti-β-Actin (mouse)	AC-15	Sigma-Aldrich	Cat# A5441, RRID:AB_476744	WB: 1:10,000-1:40,000

Journal of Cell Science • Supplementary information

**Antibody**	**Conjugate**	**Source**	**Identifier**
Anti-chicken IgY (donkey)	Alexa Fluor 647	Jackson ImmunoResearch Labs	Cat# 703-606-155, RRID:AB_2340380
Anti-chicken IgY (donkey)	Cy2	Jackson ImmunoResearch Labs	Cat# 703-225-155, RRID:AB_2340370
Anti-mouse IgG (donkey)	Cy3	Jackson ImmunoResearch Labs	Cat# 715-165-150, RRID:AB_2340813
Anti-rabbit IgG (goat)	DyLight 405	Jackson ImmunoResearch Labs	Cat# 111-475-003, RRID:AB_2338035
Anti-mouse IgG (goat)	DyLight 405	Jackson ImmunoResearch Labs	Cat# 115-475-003, RRID:AB_2338786
Anti-mouse IgG (goat)	HRP	Sigma-Aldrich	Cat# A3682, RRID:AB_258100
Anti-rabbit IgG (goat)	HRP	Sigma-Aldrich	Cat# A6154, RRID:AB_258284
Anti-rat IgG (goat)	HRP	Sigma-Aldrich	Cat# A5795, RRID:AB_258259

Journal of Cell Science • Supplementary information

Table S2. Secondary antibodies.

Antibody	Conjugate	Source	Identifier
Anti-chicken IgY (donkey)	Alexa Fluor 647	Jackson ImmunoResearch Labs	Cat# 703-606-155, RRID:AB_2340380
Anti-chicken IgY (donkey)	Cy2	Jackson ImmunoResearch Labs	Cat# 703-225-155, RRID:AB_2340370
Anti-mouse IgG (donkey)	Cy3	Jackson ImmunoResearch Labs	Cat# 715-165-150, RRID:AB_2340813
Anti-rabbit IgG (goat)	DyLight 405	Jackson ImmunoResearch Labs	Cat# 111-475-003, RRID:AB_2338035
Anti-mouse IgG (goat)	DyLight 405	Jackson ImmunoResearch Labs	Cat# 115-475-003, RRID:AB_2338786
Anti-mouse IgG (goat)	HRP	Sigma-Aldrich	Cat# A3682, RRID:AB_258100
Anti-rabbit IgG (goat)	HRP	Sigma-Aldrich	Cat# A6154, RRID:AB_258284
Anti-rat IgG (goat)	HRP	Sigma-Aldrich	Cat# A5795, RRID:AB_258259

References

Chen, S. W., Wu, B. Y., Xu, S. P., Fan, K. X., Yan, L., Gong, Y., Wen, J. B. and Wu, D. H. (2012). Suppression of CB1 cannabinoid receptor by lentivirus mediated small interfering RNA ameliorates hepatic fibrosis in rats. *PLoS One* **7**, e50850.

Choy, R. W., Park, M., Temkin, P., Herring, B. E., Marley, A., Nicoll, R. A. and von Zastrow, M. (2014). Retromer mediates a discrete route of local membrane delivery to dendrites. *Neuron* **82**, 55-62.
