# Supplementary material for: SGIP1 binding to the α-helical H9 domain of cannabinoid receptor 1 promotes axonal surface expression
Source: J Cell Sci. 2024 Jun 12;137(11):jcs261551. doi: 10.1242/jcs.261551 (PMC11213518; doi:10.1242/jcs.261551)
Supplement: Supplementary file 1 [file joces-137-261551_review_history.pdf]

SGIP1 binding to the α-helical H9 domain of cannabinoid receptor 1 promotes axonal surface expression

Alexandra Fletcher-Jones, Ellen Spackman, Tim J. Craig, Yasuko Nakamura, Kevin A. Wilkinson and Jeremy M. Henley
DOI: 10.1242/jcs.261551

Editor: Giampietro Schiavo

Review timeline
Original submission: 17 August 2023
Editorial decision: 25 September 2023
First revision received: 22 March 2024
Accepted: 30 April 2024

Original submission

First decision letter

MS ID#: JOCES/2023/261551

MS TITLE: SGIP1 binding to the α-helical *H9* domain of cannabinoid receptor 1 promotes axonal surface expression

AUTHORS: Alexandra Fletcher-Jones, Ellen Spackman, Tim J Craig, Yasuko Nakamura, Kevin A Wilkinson, and Jeremy M Henley
ARTICLE TYPE: Research Article

We have now reached a decision on the above manuscript.

To see the reviewers' reports and a copy of this decision letter, please go to: https://submit-jcs.biologists.org and click on the 'Manuscripts with Decisions' queue in the Author Area. (Corresponding author only has access to reviews.)

As you will see, the reviewers raise a few, yet substantial,criticisms that prevent me from accepting the paper at this stage. They suggest, however, that a revised version might prove acceptable, if you can address their concerns. If you think that you can deal satisfactorily with the criticisms on revision, I would be pleased to see a revised manuscript.

Please ensure that you clearly highlight all changes made in the revised manuscript. Please avoid using 'Tracked changes' in Word files as these are lost in PDF conversion.

I should be grateful if you would also provide a point-by-point response detailing how you have dealt with the points raised by the reviewers in the 'Response to Reviewers' box. Please attend to all of the reviewers' comments. If you do not agree with any of their criticisms or suggestions please explain clearly why this is so.

Reviewer 1

Advance summary and potential significance to field

© 2024. Published by The Company of Biologists under the terms of the Creative Commons Attribution License (https://creativecommons.org/licenses/by/4.0/).

Fletcher-Jones and colleagues investigate the important question: What is the mechanism behind the polarized surface expression of CB1R in neurons? This manuscript is an interesting follow-up study of the authors' recent eLife manuscript, where the helix9-deficient CB1R mutant was introduced and its unique neuronal surface expression profile was showcased.

In their present work, they elucidate how the helix 9 region interacts with the SGIP1 protein. Previous research demonstrated the SGIP1 protein can interact with the C-terminus of CB1R, subsequently influencing its internalization.

Therefore, this discovery lends valuable insights into the partners of the CB1R protein, though I would categorize this improvement as moderate in significance.

I appreciate the hypothesis that the helix9-SGIP1 interaction governs the polarized surface expression of CB1R. The data featuring the overexpressed WT and deltaH9 mutant CB1R stands out as particularly compelling.

However, certain aspects concerning SGIP1-knockdown's impact on endogenous CB1R function need rigorous re-evaluation:

In Figure 4, the interpretation of results appears ambiguous. While the paragraph title correctly mentions the alteration of CB1R's surface/total expression due to SGIP1 silencing, the subsequent analysis misrepresents this ratio's implications. With an evident increase in total CB1R expression, the actual surface expression might remain unchanged or even rise, regardless of a decreased surface/total ratio. To draw a conclusion about surface CB1 expression, the absolute level of surface CB1R should be analyzed, and it should also be presented in the figures. This has high biological importance, since the absolute level of CB1R surface expression is expected to modulate the endocannabinoid sensitivity of neurons. In agreement with my concern, the representative immunoblot shows a seemingly higher surface expression of CB1R in SGIP1-KD cells. While I agree with the authors that the KD condition possibly leads to a homeostatic increase in the total CB1 level due to the higher internalization of CB1R, the results indicate this mechanism restores the surface expression of CB1R. This likely also restores the endocannabinoid sensitivity of the system. In light of that, I am not convinced that the effect of SGIP1 knockdown on the presynaptic calcium influx is specific to CB1 receptor signaling, instead it may indicate an altered calcium handling of these neurons in the absence of SGIP1.

Comments for the author

Major Concerns:

- Figure 4 should display the absolute surface CB1R level, or alternatively normalize it against a protein unaffected by SGIP1-KD, such as GAPDH. The narrative on CB1R's surface expression in SGIP1-KD requires careful refinement.
If CB1R's surface expression doesn't decline, an explanation for the diminished response to a CB1R agonist in SGIP1-KD cells is essential.

- To validate SGIP1 silencing's specificity to CB1 signaling, it would be beneficial to prove that the response to another agonist linked with an endogenous Gi/o protein-coupled receptor (which SGIP1 doesn't interact with) remains unchanged in these neurons.

- If feasible, exploring the interaction between the full-length wild-type or deltaH9 mutant CB1 and SGIP1, beyond just CB1Rct, would be advantageous.

Minor Concerns:
- Although the main figure legends rightly present exact p-values for all statistical comparisons, these values are absent from the Supplementary Figure legends. They should be indicated.

<u>Reviewer 2</u>

Advance summary and potential significance to field

© 2024. Published by The Company of Biologists under the terms of the Creative Commons Attribution License (https://creativecommons.org/licenses/by/4.0/).

The manuscript of Fletcher-Jones et al. investigates the molecular mechanisms for the surface targeting of a prototypical axonal GPCR, the CB1 cannabinoid receptor by building on previous work of the team. Their study investigates the interaction of the protein SGIP1β, recently discovered by the Blahos lab, as a binding partner of Helix 9 (H9) of C-terminal CB1R segment, and, by modulating neuronal SGIP1β levels, show its involvement of specific H9-dependent CB1R targeting to the axonal surface. The manuscript is clearly written and illustrated and the statistics and control experiments are adequate. The finding that SGIP1β binds to the CB1R through H9 is a useful addition to the established knowledge but I believe that with some additional data, our understanding of the respective roles of SGIP1β and H9 in CB1R targeting could more comprehensive.

Comments for the author

Major concern:

Contrary to what the authors suggest in the Discussion, there is a relatively straightforward mechanical explanation to understand how the endocytosis-related SGIP1ß enhances CB1R surface expression. SGIP1 has been shown to reduce CB1R endocytosis by the Blahos team, but this has been only done in HEK cells. As neuronal sub-region specific endocytosis is a recognized element in CB1R targeting, SGIP1ß-induced modification of CB1R internalization is a plausible mechanism to regulate axonal surface targeting. In order to confirm or exclude this possibility, it would be highly interesting to measure the effect of the changes in SGIP1ß levels on CB1R endocytosis in neurons, by applying antibody-feeding method that the authors previously used (eLife, 2019). Measuring endocytosed antibody levels separately in dendrites and axons, by counting labeled endosomes, would be very informative.

Minor concerns:

1) As manipulation of SGIP1ß levels change the total quantity of CB1R, it would be useful to calculate the surface polarity index as well, not only the level of surface expression in axons and dendrites, separately.

2) Please describe in Methods the CB1R quantification on the dendritic surface shown on Fig. Suppl 2.

First revision

<u>Author response to reviewers' comments</u>

Reviewer #1:

The reviewer succinctly outlines the scope and context of our manuscript. They state that our work ‘*lends valuable insights into the partners of the CB1R*’ and that ‘*data featuring the overexpressed WT and deltaH9 mutant CB1R stands out as particularly compelling*’.

The reviewer does, however, suggest some additional experiments and further clarification of how we interpret some of our data. As detailed below and in the revised manuscript we have followed their advice, and we believe our work has been much improved as a result.

Major Concerns:

1 *Figure 4 should display the absolute surface CB1R level, or alternatively, normalize it against a protein unaffected by SGIP1-KD, such as GAPDH.*

We thank the reviewer for highlighting this important point.

GAPDH is cytosolic so it would be difficult to use this to normalise surface membrane levels of CB1R

© 2024. Published by The Company of Biologists under the terms of the Creative Commons Attribution License (https://creativecommons.org/licenses/by/4.0/).

to gain an 'absolute' measure. Rather, we have normalised CB1R against surface expression of the EGF receptor (EGFR), which we show is unaffected by SGIP1 knockdown (Figure 5G).

2 *The narrative on CB1R's surface expression in SGIP1-KD requires careful refinement. If CB1R's surface expression doesn't decline, an explanation for the diminished response to a CB1R agonist in SGIP1-KD cells is essential.*

Our new analyses, as recommended by the reviewer, show that there is indeed no difference in absolute CB1R surface levels in SGIP1 KD cells compared to SCR29. This is surprising, but it is important to note that in these experiments we analysed whole-cell surface expression across a population of neurons by surface biotinylation. Thus, while absolute CB1 surface levels are unchanged, it remains possible that perturbations in presynaptic enrichment of CB1R, as a result of SGIP1 knockdown, may lead to deficits in CB1R-mediated inhibition of Ca^{2+} signalling. Furthermore, we are mindful that work by the Blahos and Mackie groups suggests that the presence or absence of SGIP1 can have an allosteric effect on CB1R signalling, and therefore may affect signalling in the absence of overt changes in CB1R surface expression. CB1R/SGIP1 co-expression in cell lines alters CB1R- mediated, pertussis toxin-sensitive ERK1/2 phosphorylation as well as recruitment of β-arrestin and GRK3 (Gazdarica et al., 2022; Hajkova et al., 2016), whereas $G_{i/o}$ protein activation and G_q-mediated intracellular Ca^{2+} mobilisation is unaffected (Hajkova et al., 2016). We have now discussed these possibilities in our extensively revised and updated Discussion.

Importantly, however, in our revised manuscript we further demonstrate that the dynamics of CB1R endocytosis are regulated by SGIP1 (Figure 4). Thus, while acute SGIP1 knockdown can lead to compensatory changes in CB1R expression, our data are supportive of an essential role for SGIP1 in CBR1 signalling and endocytosis.

3 *To validate SGIP1 silencing's specificity to CB1 signaling, it would be beneficial to prove that the response to another agonist linked with an endogenous Gi/o protein-coupled receptor (which SGIP1 doesn't interact with) remains unchanged in these neurons.*

Again, we thank the reviewer for raising this key issue.

SGIP1 is a presynaptically-enriched cargo adaptor that could well have roles beyond regulation of CB1R surface expression. We acknowledge therefore that, while we show reduced endocannabinoid-mediated Ca^{2+} influx in response stimulation, this study does not definitively exclude the possibility that this difference could be due to altered Ca^{2+} handling, for example, the loss of voltage-gated Ca^{2+} channels themselves.

Nevertheless, previous work has suggested that SGIP1 effects can be specific and selective. For example, SGIP1 KD impairs endocytosis of synaptotagmin as measured by a synaptotagmin-pHluorin assays in response to stimulation (Lee et al., 2019). On the other hand, VAMP2-pHluorin and synaptophysin-pHluorin assays show no difference in vesicle release and endocytosis during SGIP1 KD, indicating that synaptotagmin is specifically affected by SGIP1 loss (Lee et al. 2019).

The insightful experiment suggested by the Reviewer to test Ca^{2+} influx upon activation of a different Gi/o receptor would indeed shed light on this important point. However, very few SGIP1 cargos have been identified and, to our knowledge, no presynaptic Gi/o protein-coupled receptor has been explicitly excluded as being an SGIP1 cargo.

Therefore, we would be required to screen other Gi/o protein-coupled receptors in our cultures for SGIP1 interaction though the methods we used in Figs 1-5 of the current manuscript before undertaking the suggested experiment. We believe that such an analysis is beyond the scope of the current manuscript and would be better suited to being explored in a separate publication.

We have, however, included this important point in the revised Discussion.

4 *If feasible, exploring the interaction between the full-length wild-type or deltaH9 mutant CB1 and SGIP1, beyond just CB1Rct, would be advantageous.*

© 2024. Published by The Company of Biologists under the terms of the Creative Commons Attribution License (https://creativecommons.org/licenses/by/4.0/).

We agree that this would be a good experiment, and it is one we have attempted and tried to optimise. However, it has proven extremely technically challenging. In our hands full-length CB1R expresses very poorly and is difficult to solubilize from the membrane. In consequence we have been unable to obtain full-length CB1R in the amount required to test this interaction. We note, however, that endogenous interaction of CB1R and SGIP1 has been shown previously (Hajkova et al., 2016).

Minor Concern:

> *Although the main figure legends rightly present exact p-values for all statistical comparisons, these values are absent from the Supplementary Figure legends. They should be indicated.*

We thank the reviewer for this point, and we apologise for not including this in the original manuscript. We have added the exact p-values to the Supplementary Figure legends.

Reviewer #2:

This reviewer provides a clear summary of the manuscript and states it is '*clearly written and illustrated*' and that it is a '*useful addition to the established knowledge*'.

The reviewer does, however, suggest inclusion of some additional data.

Major Concern:

1 Contrary to what the authors suggest in the Discussion, there is a relatively straightforward mechanical explanation to understand how the endocytosis- related SGIP1ß enhances CB1R surface expression. SGIP1 has been shown to reduce CB1R endocytosis by the Blahos team, but this has been only done in HEK cells. As neuronal sub-region specific endocytosis is a recognized element in CB1R targeting, SGIP1ß-induced modification of CB1R internalization is a plausible mechanism to regulate axonal surface targeting. In order to confirm or exclude this possibility, it would be highly interesting to measure the effect of the changes in SGIP1ß levels on CB1R endocytosis in neurons, by applying antibody-feeding method that the authors previously used (eLife, 2019). Measuring endocytosed antibody levels separately in dendrites and axons, by counting labelled endosomes, would be very informative.

We thank the reviewer for raising this key issue.

We have now performed new experiments (Figure 4 in the revised manuscript) to address whether the decreased proportion of surface expression of CB1R during SGIP1 knockdown is due to increased endocytosis. We used a pulse-chase strategy in which we followed the fate of surface expressed EGFP-CB1R labelled with anti- GFP antibody after incubation for 1 hr in 5 µM 2-AG to induce endocytosis under conditions where degradation of endocytosed receptors was blocked by leupeptin.
SGIP1 KD caused an increase in the accumulation of endocytosed CB1R in endolysosomes in the soma, consistent with a role for SGIP1 in maintaining CB1R surface expression and suggesting that internalised axonal receptors undergo retrograde trafficking to the soma for degradation.

Minor Concerns:

1) *As manipulation of SGIP1ß levels change the total quantity of CB1R, it would be useful to calculate the surface polarity index as well, not only the level of surface expression in axons and dendrites, separately.*

We thank the reviewer for this suggestion, and we have now included surface polarity quantification in Supplementary Figure 2 of our revised manuscript.

2) *Please describe in Methods the CB1R quantification on the dendritic surface, shown on Fig. Suppl 2.*

© 2024. Published by The Company of Biologists under the terms of the Creative Commons Attribution License (https://creativecommons.org/licenses/by/4.0/).

Again, we thank the reviewer for this point, and we apologise for not including this in the original manuscript. We have now added the relevant information to the Methods section.

References

Gazdarica, M., Noda, J., Durydivka, O., Novosadova, V., Mackie, K., Pin, J. P., Prezeau, L. and Blahos, J. (2022). SGIP1 modulates kinetics and interactions of the cannabinoid receptor 1 and G protein-coupled receptor kinase 3 signalosome. *J Neurochem* **160**, 625-642.

Hajkova, A., Techlovska, S., Dvorakova, M., Chambers, J. N., Kumpost, J., Hubalkova, P., Prezeau, L. and Blahos, J. (2016). SGIP1 alters internalization and modulates signaling of activated cannabinoid receptor 1 in a biased manner. *Neuropharmacology* **107**, 201-214.

Lee, S. E., Jeong, S., Lee, U. and Chang, S. (2019). SGIP1alpha functions as a selective endocytic adaptor for the internalization of synaptotagmin 1 at synapses. *Mol Brain* **12**, 41.

Second decision letter

MS ID#: JOCES/2023/261551

MS TITLE: SGIP1 binding to the α-helical *H9* domain of cannabinoid receptor 1 promotes axonal surface expression

AUTHORS: Alexandra Fletcher-Jones, Ellen Spackman, Tim J Craig, Yasuko Nakamura, Kevin A Wilkinson, and Jeremy M Henley
ARTICLE TYPE: Research Article

I am happy to tell you that your manuscript has been accepted for publication in the Journal of Cell Science, pending standard ethics checks.

Reviewer 1

Advance summary and potential significance to field

Fletcher-Jones et al. demonstrated that the interaction between CB1 helix9 and SGIP1 governs the polarized surface expression and internalization of CB1R in neurons, providing important mechanistic insights into the function of the CB1 receptor.

Comments for the author

The authors have addressed all of my concerns.

Reviewer 2

Advance summary and potential significance to field

The authors have added interesting data and have fully addressed my concerns.

Comments for the author

I recommend publication of the manuscript in JOCES in its current version.

© 2024. Published by The Company of Biologists under the terms of the Creative Commons Attribution License (https://creativecommons.org/licenses/by/4.0/).
